# Supplementary material for: The experience of living with patellofemoral pain—loss, confusion and fear-avoidance: a UK qualitative study
Source: BMJ Open. 2018 Jan 23;8(1):e018624. doi: 10.1136/bmjopen-2017-018624 (PMC5786111; doi:10.1136/bmjopen-2017-018624)
Supplement: Supplementary file 1 [file bmjopen-2017-018624supp001.pdf]

Data supplement 1 – Code Book

Name

Impact on self, sense of loss

Name
Pain experiences, disruption to life, distressing
Loss of identity, self
Relationships loss (time with family, friends, children, friends making fun, judgement)
Knee noises experiences, distressing

Not understanding, sense making, confusion

Name
Trying to find cause
Anatomy and imaging central to causation
Historical diagnosis – i.e historical comments still central to beliefs
Distrust of healthcare – wanting scans
Not being taken seriously
Disagreement with healthcare increases uncertainty and distress

Exercise and Activity Barriers

Name
Diagnosis uncertainty
Cultural beliefs around pain
Fear avoidance
Iatrogenic effect of healthcare
Sport = future ‘damage’
Physio = exercise
contradiction

Coping Strategies

Name
Central to coping is activity avoidance – despite saying they don’t let it stop them
Postural adjustments
Rest
Analgesics – whilst acknowledging they don’t work
Reliance on knee support – not knowing how they work

The Future

Name
Belief will get worse
Effect of healthcare on that belief (including external locus of control)
Low self-efficacy
Low opinion of physio, past physio failures
Desire to be pain free
high expectation of prognosis
