# Supplementary material for: The experience of living with patellofemoral pain—loss, confusion and fear-avoidance: a UK qualitative study
Source: BMJ Open. 2018 Jan 23;8(1):e018624. doi: 10.1136/bmjopen-2017-018624 (PMC5786111; doi:10.1136/bmjopen-2017-018624)
Supplement: Author's manuscript [file bmjopen-2017-018624.draft_revisions.pdf]

BMJ Open is committed to open peer review. As part of this commitment we make the peer review history of every article we publish publicly available.

When an article is published we post the peer reviewers' comments and the authors' responses online. We also post the versions of the paper that were used during peer review. These are the versions that the peer review comments apply to.

The versions of the paper that follow are the versions that were submitted during the peer review process. They are not the versions of record or the final published versions. They should not be cited or distributed as the published version of this manuscript.

BMJ Open is an open access journal and the full, final, typeset and author-corrected version of record of the manuscript is available on our site with no access controls, subscription charges or pay-per-view fees (http://bmjopen.bmj.com).

If you have any questions on BMJ Open's open peer review process please email editorial.bmjopen@bmj.com

BMJ Open

The lived experience of patellofemoral pain: loss, confusion and fear-avoidance

Journal:	*BMJ Open*
Manuscript ID	bmjopen-2017-018624
Article Type:	Research
Date Submitted by the Author:	11-Jul-2017
Complete List of Authors:	Smith, Benjamin; Derby Teaching Hospitals NHS Foundation Trust, Physiotherapy Outpatients; University of Nottingham, Division of Rehabilitation and Ageing, School of Medicine<br>Moffatt, Fiona; University of Nottingham, Division of Physiotherapy and Rehabilitation Sciences, School of Health Sciences,<br>Hendrick, Paul ; University of Nottingham, Division of Physiotherapy and Rehabilitation Sciences, School of Health Sciences<br>Bateman, Marcus; Derby Teaching Hospitals NHS Foundation Trust, Physiotherapy Outpatients<br>Rathleff, Michael ; Aalborg University, Research Unit for General Practice in Aalborg, Department of Clinical Medicine ; Aalborg University Hospital, Department of Occupational Therapy and Physiotherapy, Department of Clinical Medicine<br>Selfe, James; Manchester Metropolitan University, Department of Health Professions<br>Smith, Toby O.; University of East Anglia, Faculty of Medicine and Health Sciences<br>Logan, Phillipa; University of Nottingham , Division of Rehabilitation and Ageing, School of Medicine
**Primary Subject Heading**:	Qualitative research
Secondary Subject Heading:	Rehabilitation medicine, Sports and exercise medicine
Keywords:	patellofemoral pain, anterior knee pain, PFP

SCHOLARONE™
Manuscripts

The lived experience of patellofemoral pain: loss, confusion and fear-avoidance

Benjamin E Smith[1,2,*], Fiona Moffatt[3], Paul Hendrick[3], Marcus Bateman[1], Michael Skovdal Rathleff[4,5], James Selfe[6], Toby O Smith[7], Pip Logan[2]

[1]Derby Teaching Hospitals NHS Foundation Trust, Physiotherapy Department (Level 3), London Road Community Hospital, Derby DE1 2QY, UK

[2]Division of Rehabilitation and Ageing, School of Medicine, University of Nottingham, Nottingham, UK

[3]Division of Physiotherapy and Rehabilitation Sciences, School of Health Sciences, University of Nottingham, Nottingham University Hospitals (City Campus), Nottingham, UK.

[4]Research Unit for General Practice in Aalborg, Department of Clinical Medicine at Aalborg University, Aalborg, Denmark

[5]Department of Occupational Therapy and Physiotherapy, Department of Clinical Medicine, Aalborg University Hospital, Denmark

[6]Department of Health Professions, Manchester Metropolitan University, Manchester, UK

[7]Faculty of Medicine and Health Sciences, University of East Anglia, Norwich, UK

[*]Corresponding author. Tel: 01332 254631 Email: benjamin.smith3@nhs.net

Word Count: 5,500

For peer review only

Abstract

Objectives:

To investigate, through phenomenological inquiry, the lived experience and perceptions of people with patellofemoral pain, prior to starting physiotherapy.

Design:

Qualitative study design using semi-structured interviews.

Setting:

A National Health Service (NHS) physiotherapy clinic within a large UK teaching hospital.

Participants:

A convenience sample of ten participants, aged between 18 and 40, with a diagnosis of patellofemoral pain and on a physiotherapy waiting list.

Results:

Participants offered rich and detailed accounts of the impact and lived experience of patellofemoral pain, including: loss of physical and functional ability; loss of self-identity; pain related confusion and difficulty making sense of their pain; pain related fear, including fear-avoidance and 'damage' beliefs; inappropriate coping strategies and fear of the future. The five major themes that emerged from the data were: (1) impact on self; (2) uncertainty, confusion and sense making; (3) exercise and activity beliefs; (4) behavioural coping strategies and (5) expectations of the future.

Conclusions:

These findings offer an insight into the lived experience of individuals with patellofemoral pain. Previous literature has focused on pain and biomechanics, rather than the individual experience, attached meanings and any wider context within a sociocultural perspective. Our findings suggest future research is warranted into biopsychosocial targeted interventions aimed at the beliefs and pain related fear for people with patellofemoral pain. The current consensus that best-evidence treatments consisting of hip and knee strengthening may not be adequate to address the fears and beliefs identified in the current study. Further qualitative research may be warranted on the impact and interpretation of medical terminology commonly used with this patient group, for example, 'weakness' and 'patellar mal-tracking' and its impact and interpretation by patients.

Trial registration:

ISRCTN 35272486

Article Summary

Strengths and limitations of this study:

- This is the first study to use a qualitative method of inquiry to gain phenomenological data on the lived experience of people with patellofemoral pain.
- Two authors independently coded all transcripts, and a clear, transparent and reproducible methodological approach was used in the thematic analysis.

For peer review only

- For pragmatic reasons a convenience sampling technique was used. However, the study had a good representation of male and females, included a variety of ethnic groups, and had similar baseline demographics as larger NHS based studies.

For peer review only

Introduction

Patellofemoral pain (PFP) is one of the most common and costly forms of knee pain.[1–3] It has an estimated prevalence of 23% in the general population in the UK.[1] Symptoms typically include retro-patellar or diffuse peripatellar pain, aggravated by activities that load the joint, such as climbing and descending stairs, squatting and running.[4]

Historically PFP has been labelled a “benign, self-limiting condition”, that improves over time with little intervention indicated.[5] However, this belief has recently been challenged with data suggesting that the overall long-term prognosis for the majority of patients with PFP is poor.[6] Only one third of patients are pain-free one year after diagnosis,[6] and 91% still report pain and dysfunction four years post-diagnosis.[7] Quantitative data suggests that some patients withdraw from participation in physical activities,[8,9] and may develop associated psychological distress, such as fear-avoidance and catastrophising thoughts in relation to their knee pain.[10–12]

The biopsychosocial model of persistent pain has recognised that psychological factors, such as fear and catastrophising can, through changes to behaviour, modulate physiological responses to pain with the development and maintenance of persistent pain.[13–17] Psychological distress has been identified in low back pain and tendon pain populations through systematic reviews,[18,19] and qualitative methods in low back and shoulder populations,[20–22] however to our knowledge this has not been investigated in PFP. Advocates of qualitative research methods suggest that qualitative inquiry can disclose the lived experience of people with pain; and therefore be used to understand patient motivation, social engagement and provide a wealth of information about the sociocultural context to pain;[23,24] contemporary models of persistent pain have identified the importance of thinking beyond muscles and joints,[25] and qualitative inquiry can provide an insight that may lead to development of ideas and hypothesis generation within the context of the biopsychosocial model of pain. No study using qualitative methods has been published regarding PFP. Therefore the aim of this study was to give a more detailed account of the lived experience of people with PFP seeking secondary care within the UK.

For peer review only

Method

In order to address gaps in the literature this research focused on identifying themes within the participants' lived experience of PFP. Thematic analysis is the most appropriate method for this type of inquiry, as codes and themes can be created inductively to capture meaning and content without prior preconceptions allowing flexibility to generate a rich and detailed account of the data.[26]

In this study, data were analysed thematically using the guidelines set out by Braun and Clarke,[26] and was reported in line with the COnsolidated criteria for REporting Qualitative research (COREQ) checklist (see supplementary file 1).[27]

The authors took an epistemological position that recognises the experience at an individual level, and any meanings attached, whilst considering the wider context within a sociocultural perspective. Sitting central on the spectrum of realism and constructivism, this position is described as "contextualist" by Braun and Clarke.[26]

Participants

A convenience sample of ten participants with a diagnosis of PFP were recruited from an NHS physiotherapy waiting list. Based on similar studies of other musculoskeletal conditions, we anticipate this sample size would be sufficient to reach data saturation.[22,28] Participants were initially contacted by mail and followed up by a telephone call. Thirty four information sheets were sent out, and 24 potential participants were contacted by telephone; two could not make the interview before physiotherapist was due to start; five physiotherapy had already commenced; one reported resolution of symptoms; and six declined to participate. Inclusion criteria were participants aged 18 to 40 with signs and symptoms of PFP, defined as: anterior or retro-patellar pain reported on at least two of the following activities; prolonged sitting, ascending or descending stairs, squatting, jumping and running.[4] These were pre-screened during an initial telephone conversation. Exclusion criteria included: previous knee surgery; awaiting lower limb surgery; knee ligamentous instability; history of patellar dislocation; true knee locking or giving way; reasons to suspect systemic pathology, or acute illness; pregnancy or breast feeding; patellar or iliotibial tract tendinopathy; and those not able to speak or understand English.

Recruitment

Participants were offered interviews at their home, or in a hospital-based physiotherapy department; all opted to be interviewed at the hospital. On arrival the researcher (BES) introduced himself as a physiotherapist working in that department, and also a researcher conducting a PhD. The researcher explained the aims of the study. Written consent and verbal consent was taken to start recording.

Data Collection

With reference to previous literature on low back pain, shoulder pain and tendon pain,[20–22] semi-structured interviews were designed by the researchers using a topic guideline with prompts to explore participants' experience of: living with PFP; past healthcare management; their interpretation of causation of their pain; beliefs, attitudes and behaviour in relation to their pain and expectations for the future.

For peer review only

The researcher also maintained a reflective journal, noting down initial thoughts and ideas after each interview.[22] This identified that early interviews raised issues about other (past and present) musculoskeletal pain, and specific coping strategies employed by participants for their PFP. These were therefore incorporated into subsequent interview schedules.

Data Analysis

All audio files were collected and transcribed verbatim. During transcription, initial thoughts and ideas were noted in the reflective journal. Audio files were listened to several times to check for accuracy, and transcriptions were read and re-read a number of times; this initial process of data familiarisation allowed for 'data immersion' by the researchers, and generation of preliminary ideas.[26] Data coding then identified and coded pertinent features of the data giving equal priority over the whole dataset. These steps were independently conducted by two researchers (BES & FM) who met to compare codes and develop agreement on the grouping of codes into themes. The generated themes were reviewed and refined, ensuring that they explained the data in relation to the coded data, and the whole dataset. The researchers then consulted on the final two stages; themes and sub-themes were named and defined to demonstrate a clear narrative, using compelling extracts as illustrations. Consideration was given to each theme individually, but also to how they related to the dataset as a whole and other themes.[26]

Data were organised and analysed using QSR International's NVivo 11. After ten interviews, it was determined by the researchers that data saturation had occurred as no new thoughts or concepts were generated in the later interviews.

For peer review only

Results

Participants ranged from 26 to 37 years of age (mean age 30.6), with a diagnosis of PFP for a mean duration of 77 months (range: 3 months to 15 years). Seven participants were female, three were male. The interviews ranged from 13 to 43 minutes (mean time: 27 minutes).

Five themes emerged from the analysis: (1) impact on self; (2) uncertainty, confusion and sense making; (3) exercise and activity beliefs; (4) behavioural coping strategies and (5) expectations of the future. Data are presented to demonstrate the range and meaning to each theme.

Theme 1: impact on self

Participants offered rich and detailed accounts of the impact and lived experience of PFP. Loss emerged as a continuous sub-theme, and descriptions of the negative effect on their lives were broad and far-reaching. Symptoms affected their daily life, with pain being a pervasive and disruptive feature of their day, with resulting loss of physical ability:

> *"I struggle at work, bending down to get bottom shelf and getting back up, I literally have to hold onto the table to pull myself up. I can't do it off just my knees." [P7].*

> *"Yeah, well, it's a pain really because I'm walking around. I'm very stiff with that leg. Going up the stairs, down the stairs at work, getting out of a chair, getting into the car." [P6].*

In addition to loss of physical ability, loss of self and loss of self-identity was evident in the stories told by many of the participants in this study. Self and self-identity are different concepts about ways in which individuals evaluate and interpret themselves; they are nested elements that are shaped by the contexts of individual's lives, with direct influence on decisions and behaviours.[29] Self, in its broader sense, can be defined as one's individuality and process of making sense of the world around them; it is a cognitive structure that defines one's sense of worth.[30] Self-identity, however, is the cognitive structure of internalised meanings and expectations associated with one's position and role within a social network.[31]

Several participants described the negative impact of PFP on their mental well-being, with subsequent loss of self-identity:

> *"I would say the reason I got my horse was because I have mental health problems and so having a horse is my routine, structure, thing that I look forward to doing. The positive in my life. And having the knee problem makes that, makes that, not so effective. You can't do, what I imagined I would be able to do." [P4].*

Physical activity has been identified as a key quality of life domain, and the one most affected among patients with persistent pain.[32] Loss of activities for these participants included: walking; exercise; driving; holidays; time with family and friends; playing with children; duties at work and kneeling. These loss of activities directly affected participants' role and position within their social network, triggering feelings of loss of self-identity. For example, a number of participants explained how PFP affected their work, and made them question their career aspirations:

For peer review only - http://bmjopen.bmj.com/site/about/guidelines.xhtml

"I would say, it makes me like wonder, if I can do the job, not at this point but maybe when I get older and older, maybe I won't be able to do it". [P4].

Judgemental attitudes from colleagues, friends or family, were described by a number of participants, with subsequent feelings of loss of self-identity, acting as moderators to low moods and feelings of premature ageing:

"They're saying that I'm a grandma. They say, 'Yeah. If you were a horse, they'd put you down (laughter). Just joking me, but obviously, it has affected me in the way that I've had to go out of work to go over to get physio. And I have had this time off, so I don't know if they're a bit, 'Well, it's not that bad.' Because day-to-day I try to be as normal as I can." [P9].

Loss of significant relationships has emerged as a key aspect of loss in previous studies of patients with persistent pain;[33–35] and disruption to important and meaningful relationships was a strong and common theme found in patients with PFP. For example:

"I've missed out of things over the years, spending time with friends, spending time with family and that kind of thing, because I've not been able to do it." [P6].

As identified by the above extracts, PFP had a compelling and far reaching impact on the participants and their lives. The pain and its disruption to life; loss of self-identify; and loss of relationships were sub-themes that emerged from the data. These embedded sub-themes were inter-related to each other, with loss emerging as a continuous thread throughout.

Theme 2: uncertainty, confusion and sense making

Confusion and sense making formed a central part in the lives of the participants, with a strong desire from all to elucidate the cause of their pain.

"If I could find out what it was that was causing the pain, then you hope it would be gone within a year. But because we don't really know what's caused it, it's kinda trial and error. So I don't really know." [P1].

The predominant focus of the participants' beliefs and attempts at making sense of their pain was that biomechanical factors were causative, with individuals trying to link these factors to the development and maintenance of their pain.

"My running technique or, I'm not sure. I'm not sure about that. I'm not sure. I think that's one thing, maybe something to do with the running technique, or something, or something to do with that." [P8].

Furthermore, confusion was also related to the episodic nature of the symptoms, with participants attempting to relate 'flare-ups' to the same biomedical factors.

A number of participants told stories of structural and biomedical beliefs becoming deep-rooted and established when reinforced. For example, one participant recounted multiple encounters with healthcare practitioners that influenced and reinforced her structural belief.

For peer review only

> *"The work physio guy said to me that he thinks that my heels have maybe gone in which has then pulled my kneecap out of alignment. So instead of going smoothly over the joint where it's supposed to, that it's probably moving over the bone and that's the sharp pain that I'm feeling. Which did make sense because it, like I said, felt like I'd got a rock underneath my kneecap at some stage." [P9].*

Some participants remembered biomechanical focused diagnoses they had been given by a healthcare practitioner they had seen many years in the past; highlighting the power and lasting influence healthcare practitioners have on their patients. For example one participant remembered the diagnosis she had received from a healthcare practitioner over 10 years ago:

> *"I had to go to the hospital once to have x-rays… I don't know if he [doctor] was trying to scare me into doing some exercise or something, but he basically said the only thing they could do is break both of my thighs and twist them a bit and then heal them back together. And it would take me years to get back to walking properly." [P4].*

Joint noises are a common feature of normal joint movement,[22] however participants commonly reported distress and confusion at joint noises, often finding healthcare practitioners' explanations inadequate.

> *"It was the noise that was concerning me more than the pain. I'm used to hurting. I'm too small to play rugby for a start, and I'd been fighting for 20 years, so, erm, it's one of those, you get used to the pain, but it's just the noise. When you start, you sort of [say] no, that's not right." [P3].*

This was in agreement with previous research, which identified negative emotions and inaccurate etiological beliefs with joint noises in patients with PFP.[36]

Expressly linked to participants' confusion and need to find the cause of their pain was also a strong desire to pursue radiological imaging, and feelings of not being taken fully seriously by the healthcare profession when this was not forthcoming.

> *"I want to know exactly what the problem is. Obviously, the doctor said, previously going back, they said tendonitis, and now they're saying it's runner's knee or whatever. But you know, it's still like, is that 100%, are you sure that's what it is? Because I was going to ask the doctor to send me for a MRI…" [P8].*

Previous research has linked poor outcomes with radiological imaging in populations with low back pain, suggesting an over use of imaging has a detrimental effect on outcomes.[37] There was one example of the resulting radiological findings compounding the confusion and distrust, for example Participant Six explained her feelings on a normal MRI finding as:

> *"I mean I was a bit concerned, because they didn't turn around and say, you have hurt it, but it's not major but this is what you've done, but they didn't actually, they said nothing's wrong, take the knee brace off, and carry on. [I was] almost deflated, because I was like wanting to know why it was hurting, but they weren't explaining any of that to me. So it's a bit like, difficult." [P6].*

For peer review only

Another participant's story demonstrates the negative impact of discordance between healthcare practitioners' diagnosis and advice, further compounding confusion and mistrust:

> *"Well, it makes you wonder then which one to believe, because I'm like, 'Well okay, he's told me not to do anything until I'm pain-free, because he doesn't want me to aggravate it,' but when, when I came here, and obviously they said that it would probably be best to start putting an impact on it again ... " [P9].*

The sense-making processes that participants described were established from past experience of healthcare treatment, past experience of pain and cultural beliefs around structure and pain.

Theme 3: exercise and activity beliefs

All participants identified specific beliefs regarding barriers to exercise and activity. These were informed by factors relating to: diagnosis uncertainty; cultural beliefs around pain; fear-avoidant behaviours and the iatrogenic effect of healthcare.

Diagnosis uncertainty, contributed to participants' beliefs regarding exercise and activity. In particular, it underpinned a dilemma regarding the relationship between activity and potential harm:

> *"It's 'are you making it worse?' And that's the crux of it really. As I'm doing it and thinking, 'if this is hurting, should I really be doing this, or shall I pack this in and do something else?' But it's the not knowing ..." [P5].*

Cultural beliefs around pain being a direct sign of tissue damage was evident in a large proportion of the participants' narratives, resulting in negative behaviour towards exercise and activity.

> *"...with me it's always been, if something hurt it because your body's telling you if you do that you're going to cause more injury. You'll make things worse." [P6].*

Associated with the cultural beliefs on pain and damage was the resultant fear-avoidant behaviour. Participants, frequently contradicted themselves however; many participants would express the sentiment that they would not let the pain stop them from doing what they wanted to do, yet demonstrated clear fear-avoidant behaviours.

> *"So for example, we went to [holiday resort] last year; on your feet all day, walking miles and miles, I would be, like, in tears by the end of the day. I wouldn't let it stop me the next day because I would be, like, I'm doing this" [P4].*

> *"When I was in [holiday resort]; a couple of days I didn't go out and I stayed back at the hotel. Because I couldn't do it, I needed to rest." [P4].*

A predominant sub-theme was the association of sport and exercise, even in the absence of pain, as a potential precursor to future joint pain and 'damage'. Some participants attributed their current PFP to past sporting activities, despite no obvious mechanisms of injury.

For peer review only

"Another reason why I probably think this happens is because I've been very active from a very young age. Talking from the age of like eight or nine I've been involved in sports: football and cricket and badminton and whatever. I've just been all my life. And I didn't always see that as catching up with me, where that excessive amount of playing sports is having an effect on my body. [It's] going to start affecting [me] and I'm feeling it these days as I get older." [P8].

A number of participants discussed the direct impact of healthcare practitioner's advice and diagnosis labelling on their exercise and activity levels, suggesting an iatrogenic effect of healthcare for PFP patients.

"I have been told by doctors before I shouldn't run because it would jar my knee and shouldn't run or walk on an uneven surface because it will wonk my knee from side to side." [P4].

"But then when I started the physio at work and he told me that I shouldn't walk or that I shouldn't swim because he just wanted to obviously manipulate it and get me pain-free before I did anything that could possibly aggravate it. So I stopped." [P9].

This theme identified a number of beliefs associated as a barrier to activity and exercise engagement. These included diagnosis uncertainty; cultural beliefs around pain; fear-avoidant behaviours and the iatrogenic effect of healthcare.

Theme 4: behavioural coping strategies

A central coping strategy for participants of this study was the concept of rest. Many of them associated rest, and avoidance of activity, with the idea that time was necessary for the healing process, and that aggravating activities should be avoided.

"I try, obviously, sit down as much as I can." [P4].

One participant expressed an expectation that healthcare professionals would advise him not to continue with activity and exercise:

R: *So you think physios would say no [to keep physically active]?*

P8: *Physios would probably say no. Yeah, you shouldn't do it.*

Another common coping strategy was postural adjustments; participants often talked of preferred sitting positions in relation to knee flexion.

In keeping with previous research on the high levels of analgesic use in patients with PFP,[7] a common narrative shared with participants was the use of analgesics, with some acknowledging they were not effective.

"I have had some strong painkillers from the doctors. They gave me some naproxen and some codeine to manage it when it was at its worst but I try not to take them." [P9].

For peer review only

The use of knee supports was also common in the self-management strategies employed by the participants.

> *"If it hurts, it hurts. I'll try and strap my knee up. Because if I know I'm going harder in like gym classes, I'll strap my knees up before I go. And then when I get too much pain, I'll stop the exercise." [P10].*

Theme 5: expectations of the future

A number of participants expressed views, which could be contextualised as an external locus of control, with expectations of passive physiotherapeutic treatment options.

> *"I would presume manipulation of muscles groups, joints and tendons." [P3].*

Even though the majority of participants expressed negative views about the future, they all expressed a desire to be pain free, over and above any functional improvements.

> ***R:*** *With the physio, what would you class as a success?*
>
> ***P8:*** *Getting rid of the pain.*

Nine of the ten participants held negative beliefs about the future; particularly in relation to prognostic prediction following their referral to physiotherapy.

> *"But then when I'm going up the stairs and it hurts it does concern me that it's going to be every day for the rest of my life I'm going to be struggling to walk upstairs. And then I think about getting old, and I think I'm going to end up with a stair lift and living downstairs and that sort of thing." [P1].*

> *" [the pain is] definitely preying on my mind. Is it gonna stop me from going into the police, is that gonna stop me doing the things I want to do later on in life? So yeah, it does prey on my mind a little bit." [P6].*

Central to their negative beliefs about the future and their prognosis was low self-efficacy. Participants felt they had very little control over their symptoms.

> *"[In] my head, my thought process is I just hate it. Do an operation. Get rid of it. In my head, and obviously not being from the medical profession, but I'm just like, "Just get rid of the pain however it can be done." [P8].*

> *"Yes, I'm 37 now and they feel older than that. You just get that feeling, don't you, I've bounced back from lots of injuries before but this is the one that is making me think. You know, when this gets cold I can feel it, and thinking there's already arthritis there, I'm in trouble, it sets the brain going." [P3].*

For peer review only

Low expectation of physiotherapy, and past physiotherapy failed treatments were also a core theme within future expectations.

> ***R:*** *Have you got any expectations of what might happen when you walk in to see the physio?*
>
> ***P10:*** *I expect them to turn around and say physio can't help.*

> *"When I did get the physiotherapy it kinda didn't really do anything anyway. So it just made me think, it's pointless, 'cause they was trying to remove the fluid from out my knee, that like I say, made it worse to begin with. She did say your knees will feel sore, but it went back to how it was anyway, so, it just seemed like a pointless process." [P7].*

There was one exception, with one participant having positive outlook to the future and their physiotherapy referral.

> *"Oh yeah, I think it will get better. Yeah, I'd go for the better option." [P9].*

The main sub-themes that emerged under the future were: beliefs that their pain will get worse; external locus of control with regards to treatment; low self-efficacy; poor opinion of physiotherapy and previous failed physiotherapy treatments and an overwhelming desire to be pain free, over and above any practical goals for rehabilitation.

Discussion

Main Findings

Quantitative research methodologies dominate the literature for PFP. This is the first study to use a qualitative method of inquiry to gain phenomenological data on the lived experience of people with PFP. The five major themes that emerged from the data were: (1) impact on self; (2) uncertainty, confusion and sense making; (3) exercise and activity beliefs; (4) behavioural coping strategies and (5) expectations of the future.

A key finding of this study is that loss of physical ability is profound and considerable, and plays a significant role in participants' lives; despite previous research suggesting that PFP is a benign and self-limiting condition.[5] An inability to continue with significant and meaningful activities has been identified as a cause of anxiety in people with persistent pain.[38] Persistent pain interrupts behaviour and a person's self-identity by affecting a sense of who they are, and what they might become.[39] As a result, lives are socially and environmentally restricted by persistent interruptions, or an inability to complete, or even attempt important tasks and activities.[39] With changes and loss of participants' position and role, for example with employment or family duties, the internalised meanings and expectations associated with one's self-identity is further threatened.[31]

Participants expressed intense confusion around their pain and symptoms. For instance, the causative reasons were elusive and troubling, as too was the ability to predict and control the pain intensity; and any attempts that participants made at understanding were firmly within the biomechanical sphere of reasoning. An inability to make sense of pain, and the process associated

For peer review only

with sense-making and pain-related fear has been proposed in low back pain populations.[40] Previous research has identified that an inability to make sense of pain places 'lives on hold',[41] and may lead to more 'catastrophising'.[42]

There remains scientific debate and uncertainty around the underlying aetiology of PFP,[43] and there is a large variation in the way PFP is managed by physiotherapists in the UK.[44] The majority of participants in this study had previous experience of healthcare management for PFP suggesting that variation in healthcare treatment may have a negative impact on the patients' lived experience. Historically the biomedical model of pain establishes a direct relationship between tissue structure and pain,[45] and participants characteristically attributed their pain to structure and/or anatomical problems. However several studies have recently demonstrated that structural abnormalities of the patellofemoral joint on Magnetic Resonance Imaging (MRI) are not associated with PFP.[46,47] Three participants had no previous healthcare management for PFP, but nevertheless gave a biomechanical/structural cause for their pain; all three had previous physiotherapy for other pain conditions, including back, hips and ankles. This may suggest that exposure to biomechanical approaches to the management of musculoskeletal pain in general could, potentially, have a carryover to other locations of pain, with a negative effect.

The iatrogenic effect of healthcare is an emerging field of research in the low back pain population [37,48]. This study is the first to find such a theme in patients with PFP. These findings are consistent with recent research that showed that the majority of UK physiotherapists would advise their patients not to continue with exercises if they experienced any pain.[44] The fear-avoidance model of pain is a well-established with patients with persistent pain, particularly persistent low back pain;[17] additionally research has shown that fear-avoidance behaviour may also exist with clinicians.[25,44,49] The central concept of the model is cognitions and emotions that underpin fear of the pain; fears about potential physical activities exacerbating the pain and further 'damaging' bodies. The fear leads to safety seeking behaviours and hypervigilance that paradoxically maintains or exacerbates the pain and disability.[22] In contrast, if pain is perceived in a non-threatening way patients are likely to maintain physical activity levels, through which recovery can be achieved.[50,51] All of the ten participants in this study described fear-avoidant behaviour at some stage of their interview. This is the first study, which we know of, that identifies this behaviour in patients with a diagnosis of PFP.

Patellofemoral pain is often described as an 'overuse' injury,[52] and these data seem to be consistent with the patients' belief and behaviour with a definition more aligned with the English language meaning of 'overuse'. Contemporary thinking in relation to injury risk challenges the idea that PFP is simply an 'overuse' injury, with evidence suggesting that persistent and long-term under-use may be a risk factor, with consistent exposure to tissue load being considered one method of management.[53] The fear-avoidant behaviours revealed within this study would therefore be seen as negative pain behaviour, with long-term detrimental consequences.

A key finding of this research is the low expectation for the future and low self-efficacy demonstrated by the majority of the participants that could be conceptualised as 'catastrophising'. Catastrophising is conceptually within the same model of pain behaviour as fear-avoidance, with largescale overlap.[19] Low self-efficacy, fear of the future and catastrophising is a common finding in patients with persistent pain.[24,54] The National Institute of Health and Care Excellence describes pain as a complex biopsychosocial issue, associated with expectations, self-efficacy, mood and coping abilities.[55] In addition, it has been shown that self-efficacy is a strong predictor of successful

For peer review only - http://bmjopen.bmj.com/site/about/guidelines.xhtml

outcome, irrespective of the intervention delivered, for patients with persistent pain; suggesting that rehabilitation programmes for persistent musculoskeletal pain should be designed with the aim of improving self-efficacy.[56]

Clinical and research implications

This study established that a sample of patients with PFP demonstrated: pain-related fear, such as fear-avoidance; damage beliefs; difficulty with making sense of their pain; low self-efficacy and fear of the future. It may be important to address these underlying fears and beliefs with targeted therapeutic interventions; this may include an individualised biopsychosocial understanding of their pain, in conjunction with a tailored rehabilitation programme for functional restoration; such an approach has produced superior outcomes to usual treatment for persistent low back pain,[57] with promising early results in shoulder pain.[58]

The current consensus that best evidence treatments consisting of hip and knee strengthening may not be adequate to address the fears and beliefs identified in the current study. Future studies are needed to explore biopsychosocial targeted interventions for this population, particularly in relation to pain experienced by patients during exercise, followed by efficacy and effectiveness trials. Interventions may be patient education packages and self-management strategies targeting self-efficacy and physical activity. Furthermore, future qualitative work will be beneficial to understand the role of medical terminology commonly used with this patient group, for example, 'weakness' and 'patellar mal-tracking',[44] and its impact and interpretation by patients.

Study limitations and strengths

Two authors independently coded all transcripts, and this study employed a clear, transparent and reproducible methodological approach to data analysis. The authors make it clear that their clinical and research experience lie within the biopsychosocial framework of musculoskeletal pain and this study forms part of a larger body of research looking at pain education, self-management strategies and exercise interventions for individuals with PFP.[59] It is worth noting that the interviewer made it explicit to the participants that he was a physiotherapist; indeed a number of them did proceed to ask clinical questions about their condition, highlighting a power dynamic between the interviewer and participant. This may, is part, have influenced their responses.

The main limitation of this study is that for pragmatic reasons a convenience sampling technique was used. It is possible that this sample may differ from other samples within the UK, and how representative these findings are to the greater population of individuals with PFP is unknown. A purposive sampling technique may have better represented sociodemographic groups, or targeted identifiable subgroups. However, it is worth noting that the sample in this study had a good representation of male and females, and included a variety of ethnic groups; additionally, population age, gender and duration of symptoms were similar to larger NHS based studies on PFP;[60] it is therefore questionable how different the data would be.

Conclusion

These findings offer an insight into the lived experience of individuals with PFP. Previous literature have focused on pain and biomechanics, rather than the individual experience, attached meanings and any wider context within a sociocultural perspective. The participants provided rich and detailed

For peer review only

narratives of loss of physical and functional ability; loss of self-identity; pain related confusion and difficulty making sense of their pain; pain-related fear, including fear-avoidance and 'damage' beliefs; inappropriate coping strategies and fear of the future. Our findings suggest future research is warranted into biopsychosocial targeted interventions and the impact and interpretation of medical terminology.

Authors' contributions

BES was responsible for conception and design, compiling the interview schedule, interviewing, transcribing, coding, analysis and interpretation, drafting and revising the manuscript. FM was responsible for conception and design, compiling the interview schedule, coding, analysis and interpretation, drafting and revising the manuscript. PH, MB, JS, MR, TS and PL were involved in conception and design, interpretation and reviewing revisions to the manuscript. All authors have read and approved of the final manuscript.

Funding

This report is independent research arising from a Clinical Doctoral Research Fellowship, Benjamin Smith, ICA-CDRF-2015-01-002 supported by the National Institute for Health Research (NIHR) and Health Education England (HEE). The views expressed in this publication are those of the author(s) and not necessarily those of the NHS, the NIHR, HEE or the Department of Health.

Competing interests

The authors declare that they have no competing interests.

Ethics approval

This study was approved by the West Midlands - Black Country Research Ethics Committee (16/WM/0414).

Availability of data

Quotations and further details are available from Benjamin Smith at benjamin.smith3@nhs.net

For peer review only

References

1 Dey P, Callaghan M, Cook N, *et al.* A questionnaire to identify patellofemoral pain in the community: an exploration of measurement properties. *BMC Musculoskelet Disord* 2016;17:237. doi:10.1186/s12891-016-1097-5

2 Urwin M, Symmons D, Allison T, *et al.* Estimating the burden of musculoskeletal disorders in the community: the comparative prevalence of symptoms at diVerent anatomical sites, and the relation to social deprivation. *Ann Rheum Dis* 1998;57:649–55.

3 Jordan KP, Kadam UT, Hayward R, *et al.* Annual consultation prevalence of regional musculoskeletal problems in primary care: an observational study. *BMC Musculoskelet Disord* 2010;11:144. doi:10.1186/1471-2474-11-144

4 Crossley KM, Stefanik JJ, Selfe J, *et al.* 2016 Patellofemoral pain consensus statement from the 4th International Patellofemoral Pain Research Retreat, Manchester. Part 1: Terminology, definitions, clinical examination, natural history, patellofemoral osteoarthritis and patient-reported outcome m. *Br J Sports Med* 2016;50:839–43.

5 Sandow MJ, Goodfellow JW. The natural history of anterior knee pain in adolescents. *J Bone Joint Surg Br* 1985;67:36–8.

6 Collins NJ, Bierma-Zeinstra SMA, Crossley KM, *et al.* Prognostic factors for patellofemoral pain: a multicentre observational analysis. *Br J Sports Med* 2013;47:227–33. doi:10.1136/bjsports-2012-091696

7 Stathopulu E, Baildam E. Anterior knee pain: a long-term follow-up. *Rheumatology (Oxford)* 2003;42:380–2.

8 Rathleff MS, Rasmussen S, Olesen JL. Unsatisfactory long-term prognosis of conservative treatment of patellofemoral pain syndrome. *Ugeskr Laeger* 2012;174:1008–13.

9 Smith BE, Hendrick P, Logan P. Patellofemoral pain: Challenging current practice - A case report. *Man Ther* 2016;22:216–9. doi:10.1016/j.math.2015.09.002

10 Doménech J, Sanchis-Alfonso V, López L, *et al.* Influence of kinesiophobia and catastrophizing on pain and disability in anterior knee pain patients. *Knee Surgery, Sport Traumatol Arthrosc* 2013;21:1562–8. doi:10.1007/s00167-012-2238-5

11 Piva SR, Fitzgerald GK, Wisniewski S, *et al.* Predictors of pain and function outcome after rehabilitation in patients with patellofemoral pain syndrome. *J Rehabil Med* 2009;41:604–12. doi:10.2340/16501977-0372

12 Piva SR, Fitzgerald GK, Irrgang JJ, *et al.* Associates of Physical Function and Pain in Patients with Patellofemoral Pain Syndrome. *Arch Phys Med Rehabil* 2009;90:285–95. doi:10.1016/j.apmr.2008.08.214

13 Quartana PJ, Campbell CM, Edwards RR. Pain catastrophizing: a critical review. *Expert Rev Neurother* 2009;9:745–58. doi:10.1586/ern.09.34

14 Moseley GL, Arntz A. The context of a noxious stimulus affects the pain it evokes. *PAIN®* 2007;133:64–71.

15 Harvie DS, Broecker M, Smith RT, *et al.* Bogus visual feedback alters onset of movement-evoked pain in people with neck pain. *Psychol Sci* 2015;:956797614563339.

For peer review only

16 Lobanov O V, Zeidan F, McHaffie JG, *et al.* From cue to meaning: Brain mechanisms supporting the construction of expectations of pain. *PAIN®* 2014;155:129–36.

17 Chou R, Shekelle P. Will this patient develop persistent disabling low back pain? *JAMA* 2010;303:1295–302. doi:10.1001/jama.2010.344

18 Mallows A, Debenham J, Walker T, *et al.* Association of psychological variables and outcome in tendinopathy: a systematic review. *Br J Sports Med* 2016;:bjsports-2016.

19 Wertli MM, Rasmussen-Barr E, Weiser S, *et al.* The role of fear avoidance beliefs as a prognostic factor for outcome in patients with nonspecific low back pain: a systematic review. *spine J* 2014;14:816–36.

20 Osborn M, Smith JA. The personal experience of chronic benign lower back pain: An interpretative phenomenological analysis. *Br J Pain* 2015;9:65–83.

21 Smith JA, Osborn M. Pain as an assault on the self: An interpretative phenomenological analysis of the psychological impact of chronic benign low back pain. *Psychol Heal* 2007;22:517–34.

22 Jones S, Hanchard N, Hamilton S, *et al.* A qualitative study of patients' perceptions and priorities when living with primary frozen shoulder. *BMJ Open* 2013;3:e003452.

23 Walker J, Holloway I, Sofaer B. In the system: the lived experience of chronic back pain from the perspectives of those seeking help from pain clinics. *Pain* 1999;80:621–8.

24 Walker J, Sofaer B, Holloway I. The experience of chronic back pain: accounts of loss in those seeking help from pain clinics. *Eur J Pain* 2006;10:199.

25 Nijs J, Roussel N, Paul van Wilgen C, *et al.* Thinking beyond muscles and joints: Therapists' and patients' attitudes and beliefs regarding chronic musculoskeletal pain are key to applying effective treatment. *Man Ther* 2013;18:96–102.

26 Braun V, Clarke V. Using thematic analysis in psychology. *Qual Res Psychol* 2006;3:77–101.

27 Tong A, Sainsbury P, Craig J. Consolidated criteria for reporting qualitative research (COREQ): a 32-item checklist for interviews and focus groups. *Int J Qual Heal care* 2007;19:349–57.

28 Mc Auliffe S, Synott A, Casey H, *et al.* Beyond the tendon: Experiences and perceptions of people with persistent Achilles tendinopathy. *Musculoskelet Sci Pract* 2017;29:108–14.

29 Leary MR, Tangney JP. *Handbook of self and identity*. Guilford Press 2011.

30 Baumeister RF. *The self in social psychology*. 1999.

31 Stryker S, Burke PJ. The past, present, and future of an identity theory. *Soc Psychol Q* 2000;:284–97.

32 Lamé IE, Peters ML, Vlaeyen JWS, *et al.* Quality of life in chronic pain is more associated with beliefs about pain, than with pain intensity. *Eur J Pain* 2005;9:15–24.

33 Aldrich S, Eccleston C. Making sense of everyday pain. *Soc Sci Med* 2000;50:1631–41.

34 Charmaz K. From the' sick role' to stories of self: Understanding the self in illness. *Rutgers Ser Self Soc Identity* 1999;2:209–39.

For peer review only

35 Johansson EE, Hamberg K, Westman G, *et al.* The meanings of pain: an exploration of women's descriptions of symptoms. *Soc Sci Med* 1999;48:1791–802.

36 Robertson CJ, Hurley M, Jones F. People's beliefs about the meaning of crepitus in patellofemoral pain and the impact of these beliefs on their behaviour: A qualitative study. *Musculoskelet Sci Pract* 2017;28:59–64. doi:10.1016/j.msksp.2017.01.012

37 Webster BS, Bauer AZ, Choi Y, *et al.* Iatrogenic consequences of early magnetic resonance imaging in acute, work-related, disabling low back pain. *Spine (Phila Pa 1976)* 2013;38:1939–46.

38 Roy R. *Chronic pain, loss and suffering*. University of Toronto Press 2004.

39 Harris S, Morley S, Barton SB. Role loss and emotional adjustment in chronic pain. *Pain* 2003;105:363–70.

40 Bunzli S, Smith A, Schütze R, *et al.* Beliefs underlying pain-related fear and how they evolve: a qualitative investigation in people with chronic back pain and high pain-related fear. *BMJ Open* 2015;5.

41 Bunzli S, Watkins R, Smith A, *et al.* Lives on hold: a qualitative synthesis exploring the experience of chronic low-back pain. *Clin J Pain* 2013;29:907–16.

42 van Wilgen CP, van Ittersum MW, Kaptein AA, *et al.* Illness perceptions in patients with fibromyalgia and their relationship to quality of life and catastrophizing. *Arthritis Rheum* 2008;58:3618–26.

43 Doménech J, Sanchis-Alfonso V, Espejo B. Changes in catastrophizing and kinesiophobia are predictive of changes in disability and pain after treatment in patients with anterior knee pain. *Knee Surg Sports Traumatol Arthrosc* 2014;22:2295–300. doi:10.1007/s00167-014-2968-7

44 Smith BE, Hendrick P, Bateman M, *et al.* Current Management Strategies for Patellofemoral Pain: An online survey of 99 practising UK physiotherapists. *BioMed Cent Musculoskelet Disord* 2017;18. doi:10.1186/s12891-017-1539-8

45 Doménech J, Sanchis-Alfonso V, Espejo B. Influence of Psychological Factors on Pain and Disability in Anterior Knee Pain Patients. In: *Anterior Knee Pain and Patellar Instability*. London: : Springer London 2011. 123–35. doi:10.1007/978-0-85729-507-1_9

46 van der Heijden RA, Oei EHG, Bron EE, *et al.* No Difference on Quantitative Magnetic Resonance Imaging in Patellofemoral Cartilage Composition Between Patients With Patellofemoral Pain and Healthy Controls. *Am J Sports Med* 2016;44:1172–8. doi:10.1177/0363546516632507

47 van der Heijden RA, de Kanter JLM, Bierma-Zeinstra SMA, *et al.* Structural Abnormalities on Magnetic Resonance Imaging in Patients With Patellofemoral Pain: A Cross-sectional Case-Control Study. *Am J Sports Med* 2016;44:2339–46. doi:10.1177/0363546516646107

48 Lin IB, O'Sullivan PB, Coffin JA, *et al.* Disabling chronic low back pain as an iatrogenic disorder: a qualitative study in Aboriginal Australians. *BMJ Open* 2013;3:1–8.

49 Littlewood C, Lowe A, Moore J. Rotator cuff disorders: a survey of current UK physiotherapy practice. *Shoulder Elb* 2012;4:64–71. doi:10.1111/j.1758-5740.2011.00164.x

For peer review only

50 Leeuw M, Goossens MEJB, Linton SJ, *et al.* The fear-avoidance model of musculoskeletal pain: current state of scientific evidence. *J Behav Med* 2007;30:77–94. doi:10.1007/s10865-006-9085-0

51 Vlaeyen JW, Linton SJ. Fear-avoidance and its consequences in chronic musculoskeletal pain: a state of the art. *Pain* 2000;85:317–32.

52 Herrington L, Nester C. Q-angle undervalued? The relationship between Q-angle and medio-lateral position of the patella. *Clin Biomech (Bristol, Avon)* 2004;19:1070–3. doi:10.1016/j.clinbiomech.2004.07.010

53 Gabbett TJ. The training-injury prevention paradox: should athletes be training smarter and harder? *Br J Sports Med* 2016;50:273–80. doi:10.1136/bjsports-2015-095788

54 Arnstein P, Caudill M, Mandle CL, *et al.* Self efficacy as a mediator of the relationship between pain intensity, disability and depression in chronic pain patients. *Pain* 1999;80:483–91.

55 National Institute for Clinical Excellence. Osteoarthritis: care and management. 2014.

56 Miles CL, Pincus T, Carnes D, *et al.* Can we identify how programmes aimed at promoting self-management in musculoskeletal pain work and who benefits? A systematic review of sub-group analysis within RCTs. *Eur J Pain* 2011;15. doi:10.1016/j.ejpain.2011.01.016

57 Vibe Fersum K, O'Sullivan P, Skouen JS, *et al.* Efficacy of classification-based cognitive functional therapy in patients with non-specific chronic low back pain: A randomized controlled trial. *Eur J pain* 2013;17:916–28.

58 Littlewood C, Bateman M, Brown K, *et al.* A self-managed single exercise programme versus usual physiotherapy treatment for rotator cuff tendinopathy: A randomised controlled trial (the SELF study). *Clin Rehabil* 2016;30:686–96. doi:10.1177/0269215515593784

59 Smith BE, Hendrick P, Bateman M, *et al.* Study protocol: a mixed methods feasibility study for a loaded self-managed exercise programme for patellofemoral pain. *Pilot Feasibility Stud* 2017;(In Press).

60 Selfe J, Janssen J, Callaghan M, *et al.* Are there three main subgroups within the patellofemoral pain population? A detailed characterisation study of 127 patients to help develop targeted intervention (TIPPs). *Br J Sport Med* 2016;50:873–80.

For peer review only

Consolidated criteria for reporting qualitative studies (COREQ): 32-item checklist

Developed from:
Tong A, Sainsbury P, Craig J. Consolidated criteria for reporting qualitative research (COREQ): a 32-item checklist for interviews and focus groups. *International Journal for Quality in Health Care*. 2007. Volume 19, Number 6: pp. 349 – 357

YOU MUST PROVIDE A RESPONSE FOR ALL ITEMS. ENTER N/A IF NOT APPLICABLE

No. Item	Guide questions/description	Reported on Page #
**Domain 1: Research team and reflexivity**		
Personal Characteristics		
1. Inter viewer/facilitator	Which author/s conducted the inter view or focus group?	Page 5
2. Credentials	What were the researcher's credentials? E.g. PhD, MD	Page 5
3. Occupation	What was their occupation at the time of the study?	Page 5
4. Gender	Was the researcher male or female?	Page 5
5. Experience and training	What experience or training did the researcher have?	Page 5
Relationship with participants		
6. Relationship established	Was a relationship established prior to study commencement?	Page 5
7. Participant knowledge of the interviewer	What did the participants know about the researcher? e.g. personal goals, reasons for doing the research	Page 5
8. Interviewer characteristics	What characteristics were reported about the inter viewer/facilitator? e.g. Bias, assumptions, reasons and interests in the research topic	Page 5 & 15
**Domain 2: study design**		
Theoretical framework		
9. Methodological orientation and Theory	What methodological orientation was stated to underpin the study? e.g. grounded theory, discourse analysis, ethnography, phenomenology, content analysis	Page 5
Participant selection		
10. Sampling	How were participants selected? e.g. purposive, convenience, consecutive, snowball	Page 5
11. Method of approach	How were participants approached? e.g. face-to-face, telephone, mail, email	Page 5
12. Sample size	How many participants were in the study?	Page 5

13. Non-participation	How many people refused to participate or dropped out? Reasons?	Page 5
Setting		
14. Setting of data collection	Where was the data collected? e.g. home, clinic, workplace	Page 5
15. Presence of non-participants	Was anyone else present besides the participants and researchers?	Page 5
16. Description of sample	What are the important characteristics of the sample? e.g. demographic data, date	Page 7
Data collection		
17. Interview guide	Were questions, prompts, guides provided by the authors? Was it pilot tested?	Page 5
18. Repeat interviews	Were repeat inter views carried out? If yes, how many?	N/A
19. Audio/visual recording	Did the research use audio or visual recording to collect the data?	Page 5
20. Field notes	Were field notes made during and/or after the inter view or focus group?	Page 5 & 6
21. Duration	What was the duration of the inter views or focus group?	Page 7
22. Data saturation	Was data saturation discussed?	Page 6
23. Transcripts returned	Were transcripts returned to participants for comment and/or correction?	N/A
**Domain 3: analysis and findings**		
Data analysis		
24. Number of data coders	How many data coders coded the data?	Page 6
25. Description of the coding tree	Did authors provide a description of the coding tree?	N/A
26. Derivation of themes	Were themes identified in advance or derived from the data?	Page 6
27. Software	What software, if applicable, was used to manage the data?	NVivo
28. Participant checking	Did participants provide feedback on the findings?	NO
Reporting		
29. Quotations presented	Were participant quotations presented to illustrate the themes/findings? Was each quotation identified? e.g. participant number	Results
30. Data and findings consistent	Was there consistency between the data presented and the findings?	Page 13
31. Clarity of major themes	Were major themes clearly presented in the findings?	RESULTS
32. Clarity of minor themes	Is there a description of diverse cases or discussion of minor themes?	Discussion

Once you have completed this checklist, please save a copy and upload it as part of your submission. When requested to do so as part of the upload process, please select the file type: *Checklist*. You will NOT be able to proceed with

For peer review only

submission unless the checklist has been uploaded. Please DO NOT include this checklist as part of the main manuscript document. It must be uploaded as a separate file.

For peer review only

BMJ Open

The experience of living with patellofemoral pain: loss, confusion and fear-avoidance

Journal:	*BMJ Open*
Manuscript ID	bmjopen-2017-018624.R1
Article Type:	Research
Date Submitted by the Author:	24-Oct-2017
Complete List of Authors:	Smith, Benjamin; Derby Teaching Hospitals NHS Foundation Trust, Physiotherapy Outpatients; University of Nottingham, Division of Rehabilitation and Ageing, School of Medicine<br>Moffatt, Fiona; University of Nottingham, Division of Physiotherapy and Rehabilitation Sciences, School of Health Sciences,<br>Hendrick, Paul ; University of Nottingham, Division of Physiotherapy and Rehabilitation Sciences, School of Health Sciences<br>Bateman, Marcus; Derby Teaching Hospitals NHS Foundation Trust, Physiotherapy Outpatients<br>Rathleff, Michael ; Aalborg University, Research Unit for General Practice in Aalborg, Department of Clinical Medicine ; Aalborg University Hospital, Department of Occupational Therapy and Physiotherapy, Department of Clinical Medicine<br>Selfe, James; Manchester Metropolitan University, Department of Health Professions<br>Smith, Toby O.; University of East Anglia, Faculty of Medicine and Health Sciences<br>Logan, Phillipa; University of Nottingham , Division of Rehabilitation and Ageing, School of Medicine
**Primary Subject Heading**:	Qualitative research
Secondary Subject Heading:	Rehabilitation medicine, Sports and exercise medicine
Keywords:	patellofemoral pain, anterior knee pain, PFP

SCHOLARONE™
Manuscripts

The experience of living with patellofemoral pain: loss, confusion and fear-avoidance

Benjamin E Smith[1,2,*], Fiona Moffatt[3], Paul Hendrick[3], Marcus Bateman[1], Michael Skovdal Rathleff[4,5], James Selfe[6], Toby O Smith[7], Pip Logan[2]

[1]Derby Teaching Hospitals NHS Foundation Trust, Physiotherapy Department (Level 3), London Road Community Hospital, Derby DE1 2QY, UK

[2]Division of Rehabilitation and Ageing, School of Medicine, University of Nottingham, Nottingham, UK

[3]Division of Physiotherapy and Rehabilitation Sciences, School of Health Sciences, University of Nottingham, Nottingham University Hospitals (City Campus), Nottingham, UK.

[4]Research Unit for General Practice in Aalborg, Department of Clinical Medicine at Aalborg University, Aalborg, Denmark

[5]Department of Occupational Therapy and Physiotherapy, Department of Clinical Medicine, Aalborg University Hospital, Denmark

[6]Department of Health Professions, Manchester Metropolitan University, Manchester, UK

[7]Faculty of Medicine and Health Sciences, University of East Anglia, Norwich, UK

[*]Corresponding author. Tel: 01332 254631 Email: benjamin.smith3@nhs.net

For peer review only

Abstract

Objectives:

To investigate the experience of living with patellofemoral pain.

Design:

Qualitative study design using semi-structured interviews, and analysed thematically using the guidelines set out by Braun and Clarke.

Setting:

A National Health Service (NHS) physiotherapy clinic within a large UK teaching hospital.

Participants:

A convenience sample of ten participants, aged between 18 and 40, with a diagnosis of patellofemoral pain and on a physiotherapy waiting list, prior to starting physiotherapy.

Results:

Participants offered rich and detailed accounts of the impact and lived experience of patellofemoral pain, including: loss of physical and functional ability; loss of self-identity; pain related confusion and difficulty making sense of their pain; pain related fear, including fear-avoidance and 'damage' beliefs; inappropriate coping strategies and fear of the future. The five major themes that emerged from the data were: (1) impact on self; (2) uncertainty, confusion and sense making; (3) exercise and activity beliefs; (4) behavioural coping strategies and (5) expectations of the future.

Conclusions:

These findings offer an insight into the lived experience of individuals with patellofemoral pain. Previous literature has focused on pain and biomechanics, rather than the individual experience, attached meanings and any wider context within a sociocultural perspective. Our findings suggest future research is warranted into biopsychosocial targeted interventions aimed at the beliefs and pain related fear for people with patellofemoral pain. The current consensus that best-evidence treatments consisting of hip and knee strengthening may not be adequate to address the fears and beliefs identified in the current study. Further qualitative research may be warranted on the impact and interpretation of medical terminology commonly used with this patient group, for example, 'weakness' and 'patellar mal-tracking' and its impact and interpretation by patients.

Trial registration:

ISRCTN 35272486

Article Summary

Strengths and limitations of this study:

- This is the first study to use a qualitative method of inquiry on the experience of people living with patellofemoral pain.
- Two authors independently coded all transcripts, and a clear, transparent and reproducible methodological approach was used in the thematic analysis.
- For pragmatic reasons a convenience sampling technique was used.

For peer review only

Introduction

Patellofemoral pain (PFP) is one of the most common and costly forms of knee pain.[1–3] It has an estimated prevalence of 23% in the general population in the UK.[1] Symptoms typically include retro-patellar or diffuse peripatellar pain, aggravated by activities that load the joint, such as climbing and descending stairs, squatting and running.[4]

Historically PFP has been labelled a “benign, self-limiting condition”, that improves over time with little intervention indicated.[5] However, this belief has recently been challenged with data suggesting that the overall long-term prognosis for the majority of patients with PFP is poor.[6] Only one third of patients are pain-free one year after diagnosis,[6] and 91% still report pain and dysfunction four years post-diagnosis.[7] Quantitative data suggests that some patients withdraw from participation in physical activities,[8,9] and may develop associated psychological distress, such as fear-avoidance and catastrophising thoughts in relation to their knee pain.[10–12]

The biopsychosocial model of persistent pain has recognised that psychological factors, such as fear and catastrophising can, through changes to behaviour, modulate physiological responses to pain with the development and maintenance of persistent pain.[13–17] Psychological distress has been identified in low back pain and tendon pain populations through systematic reviews,[18,19] and qualitative methods in low back and shoulder populations,[20–22] however to our knowledge this has not been investigated in PFP. Advocates of qualitative research methods suggest that qualitative inquiry can disclose the experience of people with pain, and therefore be used to understand patient motivation, social engagement and provide a wealth of information about the sociocultural context to pain.[23,24] Contemporary models of persistent pain have identified the importance of thinking beyond muscles and joints,[25] and qualitative inquiry can provide an insight that may lead to development of ideas and hypothesis generation within the context of the biopsychosocial model of pain. No study using qualitative methods has been published regarding PFP. Therefore the aim of this study was to give a more detailed account of the experience of people living with PFP, seeking secondary care within the UK.

For peer review only

Method

In order to address gaps in the literature this research focused on identifying themes within the participants' experience of living with PFP. A qualitative interpretive description design was chosen as an appropriate methodological approach.[26] Thematic analysis is the most appropriate method for this type of inquiry, as codes and themes can be created inductively to capture meaning and content without prior preconceptions allowing flexibility to generate a rich and detailed account of the data.[27]

In this study, data were analysed thematically using the guidelines set out by Braun and Clarke,[27] and was reported in line with the COnsolidated criteria for REporting Qualitative research (COREQ) checklist (see supplementary file 1).[28]

Braun and Clarke[27] describe a multi-stage approach to thematic data analysis; demonstrating clear distinction of the thematic approach, whilst allowing for the inherent flexibility in the process. They reasoned that a thematic analysis can be conducted from a both realist and constructionist paradigms, although with differing outcomes. A realist approach allows theories about individual motivation and meaning to be developed, since the epistemological position is that there is a unidirectional relationship between meaning, experience and language[27]. A constructionist perspective differs, as meaning and experience are socially produced and knowledge a human and social construct; therefore theories about individual motivation and meaning are inappropriate, and theories focus instead on sociocultural contexts[27]. This study did not set out to prove or disprove a hypotheses; it set out to generate new data from which an understanding of living with PFP might be developed. The authors wanted to take an epistemological position that recognises the experience at an individual level, and any meanings attached, whilst considering the wider context within a sociocultural perspective. Sitting central on the spectrum of realism and constructivism, this position is described as "contextualist" by Braun and Clarke[27].

Recruitment

A convenience sample of ten participants with a diagnosis of PFP were recruited from an NHS physiotherapy waiting list. Based on similar studies of other musculoskeletal conditions, we anticipate this sample size would be sufficient to reach data saturation.[22,29] Participants were initially contacted by mail and followed up by a telephone call (BES). Thirty four information sheets were sent out, and 24 potential participants were contacted by telephone; two could not make the interview before physiotherapy was due to start; five people physiotherapy had already commenced; one reported resolution of symptoms; and six declined to participate. Inclusion criteria were participants aged 18 to 40 with signs and symptoms of PFP, defined as: anterior or retro-patellar pain reported on at least two of the following activities; prolonged sitting, ascending or descending stairs, squatting, jumping and running.[4] These were pre-screened during an initial telephone conversation. Exclusion criteria included: previous knee surgery; awaiting lower limb surgery; knee ligamentous instability; history of patellar dislocation; true knee locking or giving way; reasons to suspect systemic pathology, or acute illness; pregnancy or breast feeding; patellar or iliotibial tract tendinopathy; and those not able to speak or understand English. The exclusion criteria were screened prior to consent being taken (BES).

Data Collection

For peer review only - http://bmjopen.bmj.com/site/about/guidelines.xhtml

Participants were offered interviews at their home, or in a hospital-based physiotherapy department; all opted to be interviewed at the hospital. On arrival the researcher (BES) introduced himself as a physiotherapist working in that department, and also a researcher conducting a PhD. The researcher explained the aims of the study. Written consent and verbal consent was taken to start recording.

With reference to previous literature on low back pain, shoulder pain and tendon pain,[20–22] semi-structured interviews were designed by the researchers using a topic guideline with prompts to explore participants' experience of: living with PFP; past healthcare management; their interpretation of causation of their pain; beliefs, attitudes and behaviour in relation to their pain and expectations for the future. The semi-structured interviews allowed for a flexible interview, in a two-way conversation, allowing new ideas to be developed as they were brought up.

The researcher also maintained a reflective journal, noting down initial thoughts and ideas after each interview.[22] This identified that early interviews raised issues about other (past and present) musculoskeletal pain, and specific coping strategies employed by participants for their PFP. These were therefore incorporated into subsequent interview schedules.

Data Analysis

All audio files were collected and transcribed verbatim (BES). During transcription, initial thoughts and ideas were noted in the reflective journal. Audio files were listened to several times to check for accuracy, and transcriptions were read and re-read a number of times; this initial process of data familiarisation allowed for 'data immersion' by the researchers, and generation of preliminary ideas.[27] Data coding then identified and coded pertinent features of the data giving equal priority over the whole dataset. These steps were independently conducted by two researchers (BES & FM) who met to compare codes and develop agreement on the grouping of codes into themes. The generated themes were reviewed and refined, ensuring that they explained the data in relation to the coded data, and the whole dataset. The researchers then consulted on the final two stages; themes and sub-themes were named and defined to demonstrate a clear narrative, using compelling extracts as illustrations. Consideration was given to each theme individually, but also to how they related to the dataset as a whole and other themes.[27]

Data were organised and analysed using QSR International's NVivo 11. After ten interviews, it was determined by the researchers that data saturation had occurred as no new thoughts or concepts were generated in the later interviews.

For peer review only

Results

Participants ranged from 26 to 37 years of age (mean age 30.6), with a diagnosis of PFP for a mean duration of 78 months (range: 3 months to 16 years). For participants' characteristics see table 1. The interviews ranged from 13 to 43 minutes (mean time: 27 minutes).

Table 1 Characteristics of participants

Participant Number	Gender	Age	Duration of symptoms (m)	Type of Employment
1	F	26	60	Healthcare Worker
2	M	33	60	Builder
3	M	37	8	Office worker
4	F	26	192	Healthcare Worker
5	F	34	36	Office worker
6	F	27	84	Waitress
7	F	28	120	Technician
8	M	29	36	Office worker
9	F	36	3	Office worker
10	F	30	180	Office worker

F, female; M, male; m, months

The first theme that emerged from the data, impact on self, describes the participants' sense of loss, in relation to their self and self-identity. The further themes that emerged describe how the participants deal with this loss in a climate of uncertainty, how they understand or make decisions regarding exercise/activity and pain management, and how they prognosticate for the future. Data are presented to demonstrate the range and meaning to each theme.

Theme 1: impact on self

Participants offered rich and detailed accounts of the impact and lived experience of PFP. Loss of self and loss of self-identity was evident in the stories told by many of the participants in this study. Self and self-identity are different concepts about ways in which individuals evaluate and interpret themselves; they are nested elements that are shaped by the contexts of individual's lives, with direct influence on decisions and behaviours.[30] Self, in its broader sense, can be defined as one's individuality and process of making sense of the world around them; it is a cognitive structure that defines one's sense of worth.[31] Self-identity, however, is the cognitive structure of internalised meanings and expectations associated with one's position and role within a social network.[32]

Symptoms affected all participants' daily life, with pain being a pervasive and disruptive feature of their day, with resulting loss of physical ability:

> *"I struggle at work, bending down to get bottom shelf and getting back up, I literally have to hold onto the table to pull myself up. I can't do it off just my knees." [P7].*

For peer review only

"Yeah, well, it's a pain really because I'm walking around. I'm very stiff with that leg. Going up the stairs, down the stairs at work, getting out of a chair, getting into the car." [P6].

Several participants described the negative impact of PFP on their mental well-being, with subsequent loss of self-identity:

"I would say the reason I got my horse was because I have mental health problems and so having a horse is my routine, structure, thing that I look forward to doing. The positive in my life. And having the knee problem makes that, makes that, not so effective. You can't do, what I imagined I would be able to do." [P4].

Physical activity has been identified as a key quality of life domain, and the one most affected among patients with persistent pain.[33] Loss of activities for these participants included: walking; exercise; driving; holidays; time with family and friends; playing with children; duties at work and kneeling. These loss of activities directly affected participants' role and position within their social network, triggering feelings of loss of self-identity. For example, a number of participants explained how PFP affected their work, and made them question their career aspirations:

"I would say, it makes me like wonder, if I can do the job, not at this point but maybe when I get older and older, maybe I won't be able to do it". [P4].

Judgemental attitudes from colleagues, friends or family, were described by a number of participants, with subsequent feelings of loss of self-identity, acting as moderators to low moods and feelings of premature ageing:

"They're saying that I'm a grandma. They say, 'Yeah. If you were a horse, they'd put you down (laughter). Just joking me, but obviously, it has affected me in the way that I've had to go out of work to go over to get physio. And I have had this time off, so I don't know if they're a bit, 'Well, it's not that bad.' Because day-to-day I try to be as normal as I can." [P9].

Loss of significant relationships has emerged as a key aspect of loss in previous studies of patients with persistent pain;[34–36] and disruption to important and meaningful relationships was a strong and common theme found in patients with PFP. For example:

"I've missed out of things over the years, spending time with friends, spending time with family and that kind of thing, because I've not been able to do it." [P6].

As identified by the above extracts, PFP had a compelling and far reaching impact on the participants and their lives. The pain and its disruption to life; loss of self-identify; and loss of relationships were themes that emerged from the data.

Theme 2: uncertainty, confusion and sense making

Confusion and sense making formed a central part in the lives of the participants, with a strong desire from all to elucidate the cause of their pain.

For peer review only

> *"If I could find out what it was that was causing the pain, then you hope it would be gone within a year. But because we don't really know what's caused it, it's kinda trial and error. So I don't really know." [P1].*

The predominant focus of the participants' beliefs and attempts at making sense of their pain was that biomechanical factors were causative, with individuals trying to link these factors to the development and maintenance of their pain.

> *"My running technique or, I'm not sure. I'm not sure about that. I'm not sure. I think that's one thing, maybe something to do with the running technique, or something, or something to do with that." [P8].*

Furthermore, confusion was also related to the episodic nature of the symptoms, with participants attempting to relate 'flare-ups' to the same biomedical factors.

A number of participants told stories of structural and biomedical beliefs becoming deep-rooted and established when reinforced. For example, one participant recounted multiple encounters with healthcare practitioners that influenced and reinforced her structural belief.

> *"The work physio guy said to me that he thinks that my heels have maybe gone in which has then pulled my kneecap out of alignment. So instead of going smoothly over the joint where it's supposed to, that it's probably moving over the bone and that's the sharp pain that I'm feeling. Which did make sense because it, like I said, felt like I'd got a rock underneath my kneecap at some stage." [P9].*

Some participants remembered biomechanical focused diagnoses they had been given by a healthcare practitioner they had seen many years in the past; highlighting the power and lasting influence healthcare practitioners have on their patients. For example one participant remembered the diagnosis she had received from a healthcare practitioner over 10 years ago:

> *"I had to go to the hospital once to have x-rays… I don't know if he [doctor] was trying to scare me into doing some exercise or something, but he basically said the only thing they could do is break both of my thighs and twist them a bit and then heal them back together. And it would take me years to get back to walking properly." [P4].*

Joint noises are a common feature of normal joint movement,[22] however participants commonly reported distress and confusion at joint noises, often finding healthcare practitioners' explanations inadequate.

> *"It was the noise that was concerning me more than the pain. I'm used to hurting. I'm too small to play rugby for a start, and I'd been fighting for 20 years, so, erm, it's one of those, you get used to the pain, but it's just the noise. When you start, you sort of [say] no, that's not right." [P3].*

This was in agreement with previous research, which identified negative emotions and inaccurate etiological beliefs with joint noises in patients with PFP.[37]

For peer review only

Expressly linked to participants' confusion and need to find the cause of their pain was also a strong desire to pursue radiological imaging, and feelings of not being taken fully seriously by the healthcare profession when this was not forthcoming.

> *"I want to know exactly what the problem is. Obviously, the doctor said, previously going back, they said tendonitis, and now they're saying it's runner's knee or whatever. But you know, it's still like, is that 100%, are you sure that's what it is? Because I was going to ask the doctor to send me for a MRI..." [P8].*

Previous research has linked poor outcomes with radiological imaging in populations with low back pain, suggesting an over use of imaging has a detrimental effect on outcomes.[38] There was one example of the resulting radiological findings compounding the confusion and distrust, for example Participant Six explained her feelings on a normal MRI finding as:

> *"I mean I was a bit concerned, because they didn't turn around and say, you have hurt it, but it's not major but this is what you've done, but they didn't actually, they said nothing's wrong, take the knee brace off, and carry on. [I was] almost deflated, because I was like wanting to know why it was hurting, but they weren't explaining any of that to me. So it's a bit like, difficult." [P6].*

Another participant's story demonstrates the negative impact of discordance between healthcare practitioners' diagnosis and advice, further compounding confusion and mistrust:

> *"Well, it makes you wonder then which one to believe, because I'm like, 'Well okay, he's told me not to do anything until I'm pain-free, because he doesn't want me to aggravate it,' but when, when I came here, and obviously they said that it would probably be best to start putting an impact on it again … " [P9].*

The sense-making processes that participants described were established from past experience of healthcare treatment, past experience of pain and cultural beliefs around structure and pain.

Theme 3: exercise and activity beliefs

All participants identified specific beliefs regarding barriers to exercise and activity. These were informed by factors relating to: diagnosis uncertainty; cultural beliefs around pain; fear-avoidant behaviours and the iatrogenic effect of healthcare.

Diagnosis uncertainty, contributed to participants' beliefs regarding exercise and activity. In particular, it underpinned a dilemma regarding the relationship between activity and potential harm:

> *"It's 'are you making it worse?' And that's the crux of it really. As I'm doing it and thinking, 'if this is hurting, should I really be doing this, or shall I pack this in and do something else?' But it's the not knowing …" [P5].*

Cultural beliefs around pain being a direct sign of tissue damage was evident in a large proportion of the participants' narratives, resulting in negative behaviour towards exercise and activity.

> *"…with me it's always been, if something hurt it because your body's telling you if you do that you're going to cause more injury. You'll make things worse." [P6].*

For peer review only

Associated with the cultural beliefs on pain and damage was the resultant fear-avoidant behaviour. Participants, frequently contradicted themselves however; many participants would express the sentiment that they would not let the pain stop them from doing what they wanted to do, yet demonstrated clear activity withdrawal.

"So for example, we went to [holiday resort] last year; on your feet all day, walking miles and miles, I would be, like, in tears by the end of the day. I wouldn't let it stop me the next day because I would be, like, I'm doing this" [P4].

"When I was in [holiday resort]; a couple of days I didn't go out and I stayed back at the hotel. Because I couldn't do it, I needed to rest." [P4].

A predominant sub-theme was the association of sport and exercise, even in the absence of pain, as a potential precursor to future joint pain and 'damage'. Some participants attributed their current PFP to past sporting activities, despite no obvious mechanisms of injury.

"Yeah. Obviously it stems from doing long distance running." [P7].

A number of participants discussed the direct impact of healthcare practitioner's advice and diagnosis labelling on their exercise and activity levels, suggesting an iatrogenic effect of healthcare for PFP patients.

"I have been told by doctors before I shouldn't run because it would jar my knee and shouldn't run or walk on an uneven surface because it will wonk my knee from side to side." [P4].

"But then when I started the physio at work and he told me that I shouldn't walk or that I shouldn't swim because he just wanted to obviously manipulate it and get me pain-free before I did anything that could possibly aggravate it. So I stopped." [P9].

This theme identified a number of beliefs associated as a barrier to activity and exercise engagement. These included diagnosis uncertainty; cultural beliefs around pain; fear-avoidant behaviours and the iatrogenic effect of healthcare.

Theme 4: behavioural coping strategies

A central coping strategy for participants of this study was the concept of rest. Many of them associated rest, and avoidance of activity, with the idea that time was necessary for the healing process, and that aggravating activities should be avoided.

"I try, obviously, sit down as much as I can." [P4].

One participant expressed an expectation that healthcare professionals would advise him not to continue with activity and exercise:

***R:** So you think physios would say no [to keep physically active]?*

For peer review only

P8: Physios would probably say no. Yeah, you shouldn't do it.

Another common coping strategy was postural adjustments; participants often talked of preferred sitting positions in relation to knee flexion.

In keeping with previous research on the high levels of analgesic use in patients with PFP,[7] a common narrative shared with participants was the use of analgesics, with some acknowledging they were not effective.

"I have had some strong painkillers from the doctors. They gave me some naproxen and some codeine to manage it when it was at its worst but I try not to take them." [P9].

The use of knee supports was also common in the self-management strategies employed by the participants.

"If it hurts, it hurts. I'll try and strap my knee up. Because if I know I'm going harder in like gym classes, I'll strap my knees up before I go. And then when I get too much pain, I'll stop the exercise." [P10].

Theme 5: expectations of the future

A number of participants expressed views, which could be contextualised as an external locus of control, with expectations of passive physiotherapeutic treatment options.

"I would presume manipulation of muscles groups, joints and tendons." [P3].

Even though the majority of participants expressed negative views about the future, they all expressed a desire to be pain free, over and above any functional improvements.

***R:** With the physio, what would you class as a success?*

***P8:** Getting rid of the pain.*

Nine of the ten participants held negative beliefs about the future; particularly in relation to prognostic prediction following their referral to physiotherapy.

"But then when I'm going up the stairs and it hurts it does concern me that it's going to be every day for the rest of my life I'm going to be struggling to walk upstairs. And then I think about getting old, and I think I'm going to end up with a stair lift and living downstairs and that sort of thing." [P1].

" [the pain is] definitely preying on my mind. Is it gonna stop me from going into the police, is that gonna stop me doing the things I want to do later on in life? So yeah, it does prey on my mind a little bit." [P6].

Central to their negative beliefs about the future and their prognosis was low self-efficacy. Participants felt they had very little control over their symptoms.

For peer review only

"[In] my head, my thought process is I just hate it. Do an operation. Get rid of it. In my head, and obviously not being from the medical profession, but I'm just like, "Just get rid of the pain however it can be done." [P8].

"Yes, I'm 37 now and they feel older than that. You just get that feeling, don't you, I've bounced back from lots of injuries before but this is the one that is making me think. You know, when this gets cold I can feel it, and thinking there's already arthritis there, I'm in trouble, it sets the brain going." [P3].

Low expectation of physiotherapy, and past physiotherapy failed treatments were also a core theme within future expectations.

R: *Have you got any expectations of what might happen when you walk in to see the physio?*

P10: *I expect them to turn around and say physio can't help.*

"When I did get the physiotherapy it kinda didn't really do anything anyway. So it just made me think, it's pointless, 'cause they was trying to remove the fluid from out my knee, that like I say, made it worse to begin with. She did say your knees will feel sore, but it went back to how it was anyway, so, it just seemed like a pointless process." [P7].

There was one exception, with one participant having positive outlook to the future and their physiotherapy referral.

"Oh yeah, I think it will get better. Yeah, I'd go for the better option." [P9].

The main sub-themes that emerged under the future were: beliefs that their pain will get worse; external locus of control with regards to treatment; low self-efficacy; poor opinion of physiotherapy and previous failed physiotherapy treatments and an overwhelming desire to be pain free, over and above any practical goals for rehabilitation.

Discussion

Main Findings

Quantitative research methodologies dominate the literature for PFP. This is the first study to use a qualitative method of inquiry to gain data on the experiences of people living with PFP. The five major themes that emerged from the data were: (1) impact on self; (2) uncertainty, confusion and sense making; (3) exercise and activity beliefs; (4) behavioural coping strategies and (5) expectations of the future.

A key finding of this study is that loss of physical ability is profound and considerable, and plays a significant role in participants' lives; despite previous research suggesting that PFP is a benign and

For peer review only

self-limiting condition.[5] An inability to continue with significant and meaningful activities has been identified as a cause of anxiety in people with persistent pain.[39] Persistent pain interrupts behaviour and a person's self-identity by affecting a sense of who they are, and what they might become.[40] As a result, lives are socially and environmentally restricted by persistent interruptions, or an inability to complete, or even attempt important tasks and activities.[40] With changes and loss of participants' position and role, for example with employment or family duties, the internalised meanings and expectations associated with one's self-identity is further threatened.[32]

Participants expressed intense confusion around their pain and symptoms. For instance, the causative reasons were elusive and troubling, as too was the ability to predict and control the pain intensity; and any attempts that participants made at understanding were firmly within the biomechanical sphere of reasoning. An inability to make sense of pain, and the process associated with sense-making and pain-related fear has been proposed in low back pain populations.[41] Previous research has identified that an inability to make sense of pain places 'lives on hold',[42] and may lead to more 'catastrophising'.[43]

There remains scientific debate and uncertainty around the underlying aetiology of PFP,[44] and there is a large variation in the way PFP is managed by physiotherapists in the UK.[45] The majority of participants in this study had previous experience of healthcare management for PFP suggesting that variation in healthcare treatment may have a negative impact on the patients' lived experience. Historically the biomedical model of pain establishes a direct relationship between tissue structure and pain,[46] and participants characteristically attributed their pain to structure and/or anatomical problems. However several studies have recently demonstrated that structural abnormalities of the patellofemoral joint on Magnetic Resonance Imaging (MRI) are not associated with PFP.[47,48] Three participants had no previous healthcare management for PFP, but nevertheless gave a biomechanical/structural cause for their pain; all three had previous physiotherapy for other pain conditions, including back, hips and ankles. This may suggest that exposure to biomechanical approaches to the management of musculoskeletal pain in general could, potentially, have a carryover to other locations of pain, with a negative effect.

The iatrogenic effect of healthcare is an emerging field of research in the low back pain population [38,49]. This study is the first to find such a theme in patients with PFP. These findings are consistent with recent research that showed that the majority of UK physiotherapists would advise their patients not to continue with exercises if they experienced any pain.[45] The fear-avoidance model of pain is a well-established with patients with persistent pain, particularly persistent low back pain;[17] additionally research has shown that fear-avoidance behaviour may also exist with clinicians.[25,45,50] The central concept of the model is cognitions and emotions that underpin fear of the pain; fears about potential physical activities exacerbating the pain and further 'damaging' bodies. The fear leads to safety seeking behaviours and hypervigilance that paradoxically maintains or exacerbates the pain and disability.[22] In contrast, if pain is perceived in a non-threatening way patients are likely to maintain physical activity levels, through which recovery can be achieved.[51,52] All of the ten participants in this study described fear-avoidant behaviour at some stage of their interview. This is the first study, which we know of, that identifies this behaviour in patients with a diagnosis of PFP.

Patellofemoral pain is often described as an 'overuse' injury,[53] and these data seem to be consistent with the patients' belief and behaviour with a definition more aligned with the English language meaning of 'overuse'. Contemporary thinking in relation to injury risk challenges the idea that PFP is

simply an ‘overuse’ injury, with evidence suggesting that persistent and long-term under-use may be a risk factor, with consistent exposure to tissue load being considered one method of management.[54] The fear-avoidant behaviours revealed within this study would therefore be seen as negative pain behaviour, with long-term detrimental consequences.

A key finding of this research is the low expectation for the future and low self-efficacy demonstrated by the majority of the participants that could be conceptualised as ‘catastrophising’. Catastrophising is conceptually within the same model of pain behaviour as fear-avoidance, with largescale overlap.[19] Low self-efficacy, fear of the future and catastrophising is a common finding in patients with persistent pain.[24,55] The National Institute of Health and Care Excellence describes pain as a complex biopsychosocial issue, associated with expectations, self-efficacy, mood and coping abilities.[56] In addition, it has been shown that self-efficacy is a strong predictor of successful outcome, irrespective of the intervention delivered, for patients with persistent pain; suggesting that rehabilitation programmes for persistent musculoskeletal pain should be designed with the aim of improving self-efficacy.[57]

Clinical and research implications

This study established that a sample of patients with PFP demonstrated: pain-related fear, such as fear-avoidance; damage beliefs; difficulty with making sense of their pain; low self-efficacy and fear of the future.

The current consensus that best evidence treatments consisting of hip and knee strengthening may not be adequate to address the fears and beliefs identified in the current study. Future studies are needed to explore biopsychosocial targeted interventions for this population, particularly in relation to pain experienced by patients during exercise, followed by efficacy and effectiveness trials. Interventions may be patient education packages and self-management strategies targeting self-efficacy and physical activity. Furthermore, future qualitative work will be beneficial to understand the role of medical terminology commonly used with this patient group, for example, ‘weakness’ and ‘patellar mal-tracking’,[45] and its impact and interpretation by patients.

Study limitations and strengths

Two authors independently coded all transcripts, and this study employed a clear, transparent and reproducible methodological approach to data analysis. The authors make it clear that their clinical and research experience lie within the biopsychosocial framework of musculoskeletal pain and this study forms part of a larger body of research looking at pain education, self-management strategies and exercise interventions for individuals with PFP.[58] It is worth noting that the interviewer made it explicit to the participants that he was a physiotherapist; indeed a number of them did proceed to ask clinical questions about their condition, highlighting a power dynamic between the interviewer and participant. This may, is part, have influenced their responses.

The main limitation of this study is that for pragmatic reasons a convenience sampling technique was used. It is possible that this sample may differ from other samples within the UK, and how representative these findings are to the greater population of individuals with PFP is unknown. A purposive sampling technique may have better represented sociodemographic groups, or targeted identifiable subgroups.

For peer review only

Conclusion

These findings offer an insight into the lived experience of individuals with PFP. Previous literature have focused on pain and biomechanics, rather than the individual experience, attached meanings and any wider context within a sociocultural perspective. The participants provided rich and detailed narratives of loss of physical and functional ability; loss of self-identity; pain related confusion and difficulty making sense of their pain; pain-related fear, including fear-avoidance and 'damage' beliefs; inappropriate coping strategies and fear of the future. Our findings suggest future research is warranted into biopsychosocial targeted interventions and the impact and interpretation of medical terminology.

Authors' contributions

BES was responsible for conception and design, compiling the interview schedule, interviewing, transcribing, coding, analysis and interpretation, drafting and revising the manuscript. FM was responsible for conception and design, compiling the interview schedule, coding, analysis and interpretation, drafting and revising the manuscript. PH, MB, JS, MR, TS and PL were involved in conception and design, interpretation and reviewing revisions to the manuscript. All authors have read and approved of the final manuscript.

Funding

This report is independent research arising from a Clinical Doctoral Research Fellowship, Benjamin Smith, ICA-CDRF-2015-01-002 supported by the National Institute for Health Research (NIHR) and Health Education England (HEE). The views expressed in this publication are those of the author(s) and not necessarily those of the NHS, the NIHR, HEE or the Department of Health.

Competing interests

The authors declare that they have no competing interests.

Ethics approval

This study was approved by the West Midlands - Black Country Research Ethics Committee (16/WM/0414).

Availability of data

Quotations and further details are available from Benjamin Smith at benjamin.smith3@nhs.net

For peer review only

References

1 Dey P, Callaghan M, Cook N, *et al.* A questionnaire to identify patellofemoral pain in the community: an exploration of measurement properties. *BMC Musculoskelet Disord* 2016;17:237. doi:10.1186/s12891-016-1097-5

2 Urwin M, Symmons D, Allison T, *et al.* Estimating the burden of musculoskeletal disorders in the community: the comparative prevalence of symptoms at diVerent anatomical sites, and the relation to social deprivation. *Ann Rheum Dis* 1998;57:649–55.

3 Jordan KP, Kadam UT, Hayward R, *et al.* Annual consultation prevalence of regional musculoskeletal problems in primary care: an observational study. *BMC Musculoskelet Disord* 2010;11:144. doi:10.1186/1471-2474-11-144

4 Crossley KM, Stefanik JJ, Selfe J, *et al.* 2016 Patellofemoral pain consensus statement from the 4th International Patellofemoral Pain Research Retreat, Manchester. Part 1: Terminology, definitions, clinical examination, natural history, patellofemoral osteoarthritis and patient-reported outcome m. *Br J Sports Med* 2016;50:839–43.

5 Sandow MJ, Goodfellow JW. The natural history of anterior knee pain in adolescents. *J Bone Joint Surg Br* 1985;67:36–8.

6 Collins NJ, Bierma-Zeinstra SMA, Crossley KM, *et al.* Prognostic factors for patellofemoral pain: a multicentre observational analysis. *Br J Sports Med* 2013;47:227–33. doi:10.1136/bjsports-2012-091696

7 Stathopulu E, Baildam E. Anterior knee pain: a long-term follow-up. *Rheumatology (Oxford)* 2003;42:380–2.

8 Rathleff MS, Rasmussen S, Olesen JL. Unsatisfactory long-term prognosis of conservative treatment of patellofemoral pain syndrome. *Ugeskr Laeger* 2012;174:1008–13.

9 Smith BE, Hendrick P, Logan P. Patellofemoral pain: Challenging current practice - A case report. *Man Ther* 2016;22:216–9. doi:10.1016/j.math.2015.09.002

10 Doménech J, Sanchis-Alfonso V, López L, *et al.* Influence of kinesiophobia and catastrophizing on pain and disability in anterior knee pain patients. *Knee Surgery, Sport Traumatol Arthrosc* 2013;21:1562–8. doi:10.1007/s00167-012-2238-5

11 Piva SR, Fitzgerald GK, Wisniewski S, *et al.* Predictors of pain and function outcome after rehabilitation in patients with patellofemoral pain syndrome. *J Rehabil Med* 2009;41:604–12. doi:10.2340/16501977-0372

For peer review only

12 Piva SR, Fitzgerald GK, Irrgang JJ, *et al.* Associates of Physical Function and Pain in Patients with Patellofemoral Pain Syndrome. *Arch Phys Med Rehabil* 2009;90:285–95. doi:10.1016/j.apmr.2008.08.214

13 Quartana PJ, Campbell CM, Edwards RR. Pain catastrophizing: a critical review. *Expert Rev Neurother* 2009;9:745–58. doi:10.1586/ern.09.34

14 Moseley GL, Arntz A. The context of a noxious stimulus affects the pain it evokes. *PAIN®* 2007;133:64–71.

15 Harvie DS, Broecker M, Smith RT, *et al.* Bogus visual feedback alters onset of movement-evoked pain in people with neck pain. *Psychol Sci* 2015;:0956797614563339.

16 Lobanov O V, Zeidan F, McHaffie JG, *et al.* From cue to meaning: Brain mechanisms supporting the construction of expectations of pain. *PAIN®* 2014;155:129–36.

17 Chou R, Shekelle P. Will this patient develop persistent disabling low back pain? *JAMA* 2010;303:1295–302. doi:10.1001/jama.2010.344

18 Mallows A, Debenham J, Walker T, *et al.* Association of psychological variables and outcome in tendinopathy: a systematic review. *Br J Sports Med* 2016;:bjsports – 2016.

19 Wertli MM, Rasmussen-Barr E, Weiser S, *et al.* The role of fear avoidance beliefs as a prognostic factor for outcome in patients with nonspecific low back pain: a systematic review. *spine J* 2014;14:816–36.

20 Osborn M, Smith JA. The personal experience of chronic benign lower back pain: An interpretative phenomenological analysis. *Br J Pain* 2015;9:65–83.

21 Smith JA, Osborn M. Pain as an assault on the self: An interpretative phenomenological analysis of the psychological impact of chronic benign low back pain. *Psychol Heal* 2007;22:517–34.

22 Jones S, Hanchard N, Hamilton S, *et al.* A qualitative study of patients' perceptions and priorities when living with primary frozen shoulder. *BMJ Open* 2013;3:e003452.

23 Walker J, Holloway I, Sofaer B. In the system: the lived experience of chronic back pain from the perspectives of those seeking help from pain clinics. *Pain* 1999;80:621–8.

24 Walker J, Sofaer B, Holloway I. The experience of chronic back pain: accounts of loss in those seeking help from pain clinics. *Eur J Pain* 2006;10:199.

For peer review only

25 Nijs J, Roussel N, Paul van Wilgen C, *et al.* Thinking beyond muscles and joints: Therapists' and patients' attitudes and beliefs regarding chronic musculoskeletal pain are key to applying effective treatment. *Man Ther* 2013;18:96–102.

26 Thorne S, Kirkham SR, MacDonald-Emes J. Focus on qualitative methods. Interpretive description: a noncategorical qualitative alternative for developing nursing knowledge. *Res Nurs Health* 1997;20:169–77.

27 Braun V, Clarke V. Using thematic analysis in psychology. *Qual Res Psychol* 2006;3:77–101.

28 Tong A, Sainsbury P, Craig J. Consolidated criteria for reporting qualitative research (COREQ): a 32-item checklist for interviews and focus groups. *Int J Qual Heal care* 2007;19:349–57.

29 Mc Auliffe S, Synott A, Casey H, *et al.* Beyond the tendon: Experiences and perceptions of people with persistent Achilles tendinopathy. *Musculoskelet Sci Pract* 2017;29:108–14.

30 Leary MR, Tangney JP. *Handbook of self and identity*. Guilford Press 2011.

31 Baumeister RF. *The self in social psychology*. 1999.

32 Stryker S, Burke PJ. The past, present, and future of an identity theory. *Soc Psychol Q* 2000;:284–97.

33 Lamé IE, Peters ML, Vlaeyen JWS, *et al.* Quality of life in chronic pain is more associated with beliefs about pain, than with pain intensity. *Eur J Pain* 2005;9:15–24.

34 Aldrich S, Eccleston C. Making sense of everyday pain. *Soc Sci Med* 2000;50:1631–41.

35 Charmaz K. From the' sick role' to stories of self: Understanding the self in illness. *Rutgers Ser Self Soc Identity* 1999;2:209–39.

36 Johansson EE, Hamberg K, Westman G, *et al.* The meanings of pain: an exploration of women's descriptions of symptoms. *Soc Sci Med* 1999;48:1791–802.

37 Robertson CJ, Hurley M, Jones F. People's beliefs about the meaning of crepitus in patellofemoral pain and the impact of these beliefs on their behaviour: A qualitative study. *Musculoskelet Sci Pract* 2017;28:59–64. doi:10.1016/j.msksp.2017.01.012

38 Webster BS, Bauer AZ, Choi Y, *et al.* Iatrogenic consequences of early magnetic resonance imaging in acute, work-related, disabling low back pain. *Spine (Phila Pa 1976)* 2013;38:1939–46.

For peer review only

39 Roy R. *Chronic pain, loss and suffering*. University of Toronto Press 2004.

40 Harris S, Morley S, Barton SB. Role loss and emotional adjustment in chronic pain. *Pain* 2003;105:363–70.

41 Bunzli S, Smith A, Schütze R, *et al.* Beliefs underlying pain-related fear and how they evolve: a qualitative investigation in people with chronic back pain and high pain-related fear. *BMJ Open* 2015;5.

42 Bunzli S, Watkins R, Smith A, *et al.* Lives on hold: a qualitative synthesis exploring the experience of chronic low-back pain. *Clin J Pain* 2013;29:907–16.

43 van Wilgen CP, van Ittersum MW, Kaptein AA, *et al.* Illness perceptions in patients with fibromyalgia and their relationship to quality of life and catastrophizing. *Arthritis Rheum* 2008;58:3618–26.

44 Doménech J, Sanchis-Alfonso V, Espejo BBB, *et al.* Changes in catastrophizing and kinesiophobia are predictive of changes in disability and pain after treatment in patients with anterior knee pain. *Knee Surg Sports Traumatol Arthrosc* 2014;22:2295–300. doi:10.1007/s00167-014-2968-7

45 Smith BE, Hendrick P, Bateman M, *et al.* Current Management Strategies for Patellofemoral Pain: An online survey of 99 practising UK physiotherapists. *BioMed Cent Musculoskelet Disord* 2017;18. doi:10.1186/s12891-017-1539-8

46 Doménech J, Sanchis-Alfonso V, Espejo B. Influence of Psychological Factors on Pain and Disability in Anterior Knee Pain Patients. In: *Anterior Knee Pain and Patellar Instability*. London: : Springer London 2011. 123–35. doi:10.1007/978-0-85729-507-1_9

47 van der Heijden RA, Oei EHG, Bron EE, *et al.* No Difference on Quantitative Magnetic Resonance Imaging in Patellofemoral Cartilage Composition Between Patients With Patellofemoral Pain and Healthy Controls. *Am J Sports Med* 2016;44:1172–8. doi:10.1177/0363546516632507

48 van der Heijden RA, de Kanter JLM, Bierma-Zeinstra SMA, *et al.* Structural Abnormalities on Magnetic Resonance Imaging in Patients With Patellofemoral Pain: A Cross-sectional Case-Control Study. *Am J Sports Med* 2016;44:2339–46. doi:10.1177/0363546516646107

49 Lin IB, O'Sullivan PB, Coffin JA, *et al.* Disabling chronic low back pain as an iatrogenic disorder: a qualitative study in Aboriginal Australians. *BMJ Open* 2013;3:1–8.

50 Littlewood C, Lowe A, Moore J. Rotator cuff disorders: a survey of current UK physiotherapy practice. *Shoulder Elb* 2012;4:64–71. doi:10.1111/j.1758-5740.2011.00164.x

For peer review only

51 Leeuw M, Goossens MEJB, Linton SJ, *et al.* The fear-avoidance model of musculoskeletal pain: current state of scientific evidence. *J Behav Med* 2007;30:77–94. doi:10.1007/s10865-006-9085-0

52 Vlaeyen JW, Linton SJ. Fear-avoidance and its consequences in chronic musculoskeletal pain: a state of the art. *Pain* 2000;85:317–32.

53 Herrington L, Nester C. Q-angle undervalued? The relationship between Q-angle and medio-lateral position of the patella. *Clin Biomech (Bristol, Avon)* 2004;19:1070–3. doi:10.1016/j.clinbiomech.2004.07.010

54 Gabbett TJ. The training-injury prevention paradox: should athletes be training smarter and harder? *Br J Sports Med* 2016;50:273–80. doi:10.1136/bjsports-2015-095788

55 Arnstein P, Caudill M, Mandle CL, *et al.* Self efficacy as a mediator of the relationship between pain intensity, disability and depression in chronic pain patients. *Pain* 1999;80:483–91.

56 National Institute for Clinical Excellence. Osteoarthritis: care and management. 2014.

57 Miles CL, Pincus T, Carnes D, *et al.* Can we identify how programmes aimed at promoting self-management in musculoskeletal pain work and who benefits? A systematic review of sub-group analysis within RCTs. *Eur J Pain* 2011;15. doi:10.1016/j.ejpain.2011.01.016

58 Smith BE, Hendrick P, Bateman M, *et al.* Study protocol: a mixed methods feasibility study for a loaded self-managed exercise programme for patellofemoral pain. *Pilot Feasibility Stud* 2017;4. doi:10.1186/s40814-017-0167-2

For peer review only

Consolidated criteria for reporting qualitative studies (COREQ): 32-item checklist

Developed from:
Tong A, Sainsbury P, Craig J. Consolidated criteria for reporting qualitative research (COREQ): a 32-item checklist for interviews and focus groups. *International Journal for Quality in Health Care*. 2007. Volume 19, Number 6: pp. 349 – 357

YOU MUST PROVIDE A RESPONSE FOR ALL ITEMS. ENTER N/A IF NOT APPLICABLE

No. Item	Guide questions/description	Reported on Page #
**Domain 1: Research team and reflexivity**		
Personal Characteristics		
1. Inter viewer/facilitator	Which author/s conducted the inter view or focus group?	Page 5
2. Credentials	What were the researcher's credentials? E.g. PhD, MD	Page 5
3. Occupation	What was their occupation at the time of the study?	Page 5
4. Gender	Was the researcher male or female?	Page 5
5. Experience and training	What experience or training did the researcher have?	Page 5
Relationship with participants		
6. Relationship established	Was a relationship established prior to study commencement?	Page 5
7. Participant knowledge of the interviewer	What did the participants know about the researcher? e.g. personal goals, reasons for doing the research	Page 5
8. Interviewer characteristics	What characteristics were reported about the inter viewer/facilitator? e.g. Bias, assumptions, reasons and interests in the research topic	Page 5 & 15
**Domain 2: study design**		
Theoretical framework		
9. Methodological orientation and Theory	What methodological orientation was stated to underpin the study? e.g. grounded theory, discourse analysis, ethnography, phenomenology, content analysis	Page 5
Participant selection		
10. Sampling	How were participants selected? e.g. purposive, convenience, consecutive, snowball	Page 5
11. Method of approach	How were participants approached? e.g. face-to-face, telephone, mail, email	Page 5
12. Sample size	How many participants were in the study?	Page 5

For peer review only

13. Non-participation	How many people refused to participate or dropped out? Reasons?	Page 5
Setting		
14. Setting of data collection	Where was the data collected? e.g. home, clinic, workplace	Page 5
15. Presence of non-participants	Was anyone else present besides the participants and researchers?	Page 5
16. Description of sample	What are the important characteristics of the sample? e.g. demographic data, date	Page 7
Data collection		
17. Interview guide	Were questions, prompts, guides provided by the authors? Was it pilot tested?	Page 5
18. Repeat interviews	Were repeat inter views carried out? If yes, how many?	N/A
19. Audio/visual recording	Did the research use audio or visual recording to collect the data?	Page 5
20. Field notes	Were field notes made during and/or after the inter view or focus group?	Page 5 & 6
21. Duration	What was the duration of the inter views or focus group?	Page 7
22. Data saturation	Was data saturation discussed?	Page 6
23. Transcripts returned	Were transcripts returned to participants for comment and/or correction?	N/A
**Domain 3: analysis and findings**		
Data analysis		
24. Number of data coders	How many data coders coded the data?	Page 6
25. Description of the coding tree	Did authors provide a description of the coding tree?	N/A
26. Derivation of themes	Were themes identified in advance or derived from the data?	Page 6
27. Software	What software, if applicable, was used to manage the data?	NVivo
28. Participant checking	Did participants provide feedback on the findings?	NO
Reporting		
29. Quotations presented	Were participant quotations presented to illustrate the themes/findings? Was each quotation identified? e.g. participant number	Results
30. Data and findings consistent	Was there consistency between the data presented and the findings?	Page 13
31. Clarity of major themes	Were major themes clearly presented in the findings?	RESULTS
32. Clarity of minor themes	Is there a description of diverse cases or discussion of minor themes?	Discussion

Once you have completed this checklist, please save a copy and upload it as part of your submission. When requested to do so as part of the upload process, please select the file type: *Checklist*. You will NOT be able to proceed with

For peer review only

submission unless the checklist has been uploaded. Please DO NOT include this checklist as part of the main manuscript document. It must be uploaded as a separate file.

For peer review only

BMJ Open

The experience of living with patellofemoral pain: loss, confusion and fear-avoidance – a UK qualitative study

Journal:	*BMJ Open*
Manuscript ID	bmjopen-2017-018624.R2
Article Type:	Research
Date Submitted by the Author:	16-Nov-2017
Complete List of Authors:	Smith, Benjamin; Derby Teaching Hospitals NHS Foundation Trust, Physiotherapy Outpatients; University of Nottingham, Division of Rehabilitation and Ageing, School of Medicine<br>Moffatt, Fiona; University of Nottingham, Division of Physiotherapy and Rehabilitation Sciences, School of Health Sciences,<br>Hendrick, Paul ; University of Nottingham, Division of Physiotherapy and Rehabilitation Sciences, School of Health Sciences<br>Bateman, Marcus; Derby Teaching Hospitals NHS Foundation Trust, Physiotherapy Outpatients<br>Rathleff, Michael ; Aalborg University, Research Unit for General Practice in Aalborg, Department of Clinical Medicine ; Aalborg University Hospital, Department of Occupational Therapy and Physiotherapy, Department of Clinical Medicine<br>Selfe, James; Manchester Metropolitan University, Department of Health Professions<br>Smith, Toby O.; University of East Anglia, Faculty of Medicine and Health Sciences<br>Logan, Phillipa; University of Nottingham , Division of Rehabilitation and Ageing, School of Medicine
**Primary Subject Heading**:	Qualitative research
Secondary Subject Heading:	Rehabilitation medicine, Sports and exercise medicine
Keywords:	patellofemoral pain, anterior knee pain, PFP

SCHOLARONE™
Manuscripts

The experience of living with patellofemoral pain: loss, confusion and fear-avoidance – a UK qualitative study

Benjamin E Smith[1,2,*], Fiona Moffatt[3], Paul Hendrick[3], Marcus Bateman[1], Michael Skovdal Rathleff[4,5], James Selfe[6], Toby O Smith[7], Pip Logan[2]

[1]Derby Teaching Hospitals NHS Foundation Trust, Physiotherapy Department (Level 3), London Road Community Hospital, Derby DE1 2QY, UK

[2]Division of Rehabilitation and Ageing, School of Medicine, University of Nottingham, Nottingham, UK

[3]Division of Physiotherapy and Rehabilitation Sciences, School of Health Sciences, University of Nottingham, Nottingham University Hospitals (City Campus), Nottingham, UK.

[4]Research Unit for General Practice in Aalborg, Department of Clinical Medicine at Aalborg University, Aalborg, Denmark

[5]Department of Occupational Therapy and Physiotherapy, Department of Clinical Medicine, Aalborg University Hospital, Denmark

[6]Department of Health Professions, Manchester Metropolitan University, Manchester, UK

[7]Faculty of Medicine and Health Sciences, University of East Anglia, Norwich, UK

[*]Corresponding author. Tel: 01332 254631 Email: benjamin.smith3@nhs.net

For peer review only

Abstract

Objectives:

To investigate the experience of living with patellofemoral pain.

Design:

Qualitative study design using semi-structured interviews, and analysed thematically using the guidelines set out by Braun and Clarke.

Setting:

A National Health Service (NHS) physiotherapy clinic within a large UK teaching hospital.

Participants:

A convenience sample of ten participants, aged between 18 and 40, with a diagnosis of patellofemoral pain and on a physiotherapy waiting list, prior to starting physiotherapy.

Results:

Participants offered rich and detailed accounts of the impact and lived experience of patellofemoral pain, including: loss of physical and functional ability; loss of self-identity; pain related confusion and difficulty making sense of their pain; pain related fear, including fear-avoidance and 'damage' beliefs; inappropriate coping strategies and fear of the future. The five major themes that emerged from the data were: (1) impact on self; (2) uncertainty, confusion and sense making; (3) exercise and activity beliefs; (4) behavioural coping strategies and (5) expectations of the future.

Conclusions:

These findings offer an insight into the lived experience of individuals with patellofemoral pain. Previous literature has focused on pain and biomechanics, rather than the individual experience, attached meanings and any wider context within a sociocultural perspective. Our findings suggest future research is warranted into biopsychosocial targeted interventions aimed at the beliefs and pain related fear for people with patellofemoral pain. The current consensus that best-evidence treatments consisting of hip and knee strengthening may not be adequate to address the fears and beliefs identified in the current study. Further qualitative research may be warranted on the impact and interpretation of medical terminology commonly used with this patient group, for example, 'weakness' and 'patellar mal-tracking' and its impact and interpretation by patients.

Trial registration:

ISRCTN 35272486

Article Summary

Strengths and limitations of this study:

- This is the first study to use a qualitative method of inquiry on the experience of people living with patellofemoral pain.
- Two authors independently coded all transcripts, and a clear, transparent and reproducible methodological approach was used in the thematic analysis.
- For pragmatic reasons a convenience sampling technique was used.

For peer review only

Introduction

Patellofemoral pain (PFP) is one of the most common and costly forms of knee pain.[1–3] It has an estimated prevalence of 23% in the general population in the UK.[1] Symptoms typically include retro-patellar or diffuse peripatellar pain, aggravated by activities that load the joint, such as climbing and descending stairs, squatting and running.[4]

Historically PFP has been labelled a “benign, self-limiting condition”, that improves over time with little intervention indicated.[5] However, this belief has recently been challenged with data suggesting that the overall long-term prognosis for the majority of patients with PFP is poor.[6] Only one third of patients are pain-free one year after diagnosis,[6] and 91% still report pain and dysfunction four years post-diagnosis.[7] Quantitative data suggests that some patients withdraw from participation in physical activities,[8,9] and may develop associated psychological distress, such as fear-avoidance and catastrophising thoughts in relation to their knee pain.[10–12]

The biopsychosocial model of persistent pain has recognised that psychological factors, such as fear and catastrophising can, through changes to behaviour, modulate physiological responses to pain with the development and maintenance of persistent pain.[13–17] Psychological distress has been identified in low back pain and tendon pain populations through systematic reviews,[18,19] and qualitative methods in low back and shoulder populations,[20–22] however to our knowledge this has not been investigated in PFP. Advocates of qualitative research methods suggest that qualitative inquiry can disclose the experience of people with pain, and therefore be used to understand patient motivation, social engagement and provide a wealth of information about the sociocultural context to pain.[23,24] Contemporary models of persistent pain have identified the importance of thinking beyond muscles and joints,[25] and qualitative inquiry can provide an insight that may lead to development of ideas and hypothesis generation within the context of the biopsychosocial model of pain. No study using qualitative methods has been published regarding PFP. Therefore the aim of this study was to give a more detailed account of the experience of people living with PFP, seeking secondary care within the UK.

For peer review only

Method

In order to address gaps in the literature this research focused on identifying themes within the participants' experience of living with PFP. A qualitative interpretive description design was chosen as an appropriate methodological approach.[26] Thematic analysis is the most appropriate method for this type of inquiry, as codes and themes can be created inductively to capture meaning and content without prior preconceptions allowing flexibility to generate a rich and detailed account of the data.[27]

In this study, data were analysed thematically using the guidelines set out by Braun and Clarke,[27] and was reported in line with the COnsolidated criteria for REporting Qualitative research (COREQ) checklist (see supplementary file 1).[28]

Braun and Clarke[27] describe a multi-stage approach to thematic data analysis; demonstrating clear distinction of the thematic approach, whilst allowing for the inherent flexibility in the process. They reasoned that a thematic analysis can be conducted from a both realist and constructionist paradigms, although with differing outcomes. A realist approach allows theories about individual motivation and meaning to be developed, since the epistemological position is that there is a unidirectional relationship between meaning, experience and language[27]. A constructionist perspective differs, as meaning and experience are socially produced and knowledge a human and social construct; therefore theories about individual motivation and meaning are inappropriate, and theories focus instead on sociocultural contexts[27]. This study did not set out to prove or disprove a hypotheses; it set out to generate new data from which an understanding of living with PFP might be developed. The authors wanted to take an epistemological position that recognises the experience at an individual level, and any meanings attached, whilst considering the wider context within a sociocultural perspective. Sitting central on the spectrum of realism and constructivism, this position is described as "contextualist" by Braun and Clarke[27].

Recruitment

A convenience sample of ten participants with a diagnosis of PFP were recruited from an NHS physiotherapy waiting list. Based on similar studies of other musculoskeletal conditions, we anticipated this sample size would be sufficient to reach data saturation, and was agreed a priori.[22,29] Participants were initially contacted by mail and followed up by a telephone call (BES). Thirty four information sheets were sent out, and 24 potential participants were contacted by telephone; two could not make the interview before physiotherapy was due to start; five people physiotherapy had already commenced; one reported resolution of symptoms; and six declined to participate. Inclusion criteria were participants aged 18 to 40 with signs and symptoms of PFP, defined as: anterior or retro-patellar pain reported on at least two of the following activities; prolonged sitting, ascending or descending stairs, squatting, jumping and running.[4] These were pre-screened during an initial telephone conversation. Exclusion criteria included: previous knee surgery; awaiting lower limb surgery; knee ligamentous instability; history of patellar dislocation; true knee locking or giving way; reasons to suspect systemic pathology, or acute illness; pregnancy or breast feeding; patellar or iliotibial tract tendinopathy; and those not able to speak or understand English. The exclusion criteria were screened prior to consent being taken (BES).

Data Collection

For peer review only

Participants were offered interviews at their home, or in a hospital-based physiotherapy department; all opted to be interviewed at the hospital. On arrival the researcher (BES) introduced himself as a physiotherapist working in that department, and also a researcher conducting a PhD. The researcher explained the aims of the study. Written consent and verbal consent was taken to start recording.

With reference to previous literature on low back pain, shoulder pain and tendon pain,[20–22] semi-structured interviews were designed by the researchers using a topic guideline with prompts to explore participants' experience of: living with PFP; past healthcare management; their interpretation of causation of their pain; beliefs, attitudes and behaviour in relation to their pain and expectations for the future. The semi-structured interviews allowed for a flexible interview, in a two-way conversation, allowing new ideas to be developed as they were brought up.

The researcher also maintained a reflective journal, noting down initial thoughts and ideas after each interview.[22] This identified that early interviews raised issues about other (past and present) musculoskeletal pain, and specific coping strategies employed by participants for their PFP. These were therefore incorporated into subsequent interview schedules.

Data Analysis

All audio files were collected and transcribed verbatim (BES). During transcription, initial thoughts and ideas were noted in the reflective journal. Audio files were listened to several times to check for accuracy, and transcriptions were read and re-read a number of times; this initial process of data familiarisation allowed for 'data immersion' by the researchers, and generation of preliminary ideas.[27] Data coding then identified and coded pertinent features of the data giving equal priority over the whole dataset. These steps were independently conducted by two researchers (BES & FM) who met to compare codes and develop agreement on the grouping of codes into themes. The generated themes were reviewed and refined, ensuring that they explained the data in relation to the coded data, and the whole dataset. The researchers then consulted on the final two stages; themes and sub-themes were named and defined to demonstrate a clear narrative, using compelling extracts as illustrations. Consideration was given to each theme individually, but also to how they related to the dataset as a whole and other themes.[27]

Data were organised and analysed using QSR International's NVivo 11. After ten interviews, it was determined by the researchers that data saturation had occurred as no new thoughts or concepts were generated in the later interviews.

For peer review only - http://bmjopen.bmj.com/site/about/guidelines.xhtml

Results

Participants ranged from 26 to 37 years of age (mean age 30.6), with a diagnosis of PFP for a mean duration of 78 months (range: 3 months to 16 years). For participants' characteristics see table 1. The interviews ranged from 13 to 43 minutes (mean time: 27 minutes).

Table 1 Characteristics of participants

Participant Number	Gender	Age	Duration of symptoms (m)	Type of Employment
1	F	26	60	Healthcare Worker
2	M	33	60	Builder
3	M	37	8	Office worker
4	F	26	192	Healthcare Worker
5	F	34	36	Office worker
6	F	27	84	Waitress
7	F	28	120	Technician
8	M	29	36	Office worker
9	F	36	3	Office worker
10	F	30	180	Office worker

F, female; M, male; m, months

The first theme that emerged from the data, impact on self, describes the participants' sense of loss, in relation to their self and self-identity. The further themes that emerged describe how the participants deal with this loss in a climate of uncertainty, how they understand or make decisions regarding exercise/activity and pain management, and how they prognosticate for the future. Data are presented to demonstrate the range and meaning to each theme.

Theme 1: impact on self

Participants offered rich and detailed accounts of the impact and lived experience of PFP. Loss of self and loss of self-identity was evident in the stories told by many of the participants in this study. Self and self-identity are different concepts about ways in which individuals evaluate and interpret themselves; they are nested elements that are shaped by the contexts of individual's lives, with direct influence on decisions and behaviours.[30] Self, in its broader sense, can be defined as one's individuality and process of making sense of the world around them; it is a cognitive structure that defines one's sense of worth.[31] Self-identity, however, is the cognitive structure of internalised meanings and expectations associated with one's position and role within a social network.[32]

Symptoms affected all participants' daily life, with pain being a pervasive and disruptive feature of their day, with resulting loss of physical ability:

> *"I struggle at work, bending down to get bottom shelf and getting back up, I literally have to hold onto the table to pull myself up. I can't do it off just my knees." [P7].*

For peer review only

"Yeah, well, it's a pain really because I'm walking around. I'm very stiff with that leg. Going up the stairs, down the stairs at work, getting out of a chair, getting into the car." [P6].

Several participants described the negative impact of PFP on their mental well-being, with subsequent loss of self-identity:

"I would say the reason I got my horse was because I have mental health problems and so having a horse is my routine, structure, thing that I look forward to doing. The positive in my life. And having the knee problem makes that, makes that, not so effective. You can't do, what I imagined I would be able to do." [P4].

Physical activity has been identified as a key quality of life domain, and the one most affected among patients with persistent pain.[33] Loss of activities for these participants included: walking; exercise; driving; holidays; time with family and friends; playing with children; duties at work and kneeling. These loss of activities directly affected participants' role and position within their social network, triggering feelings of loss of self-identity. For example, a number of participants explained how PFP affected their work, and made them question their career aspirations:

"I would say, it makes me like wonder, if I can do the job, not at this point but maybe when I get older and older, maybe I won't be able to do it". [P4].

Judgemental attitudes from colleagues, friends or family, were described by a number of participants, with subsequent feelings of loss of self-identity, acting as moderators to low moods and feelings of premature ageing:

"They're saying that I'm a grandma. They say, 'Yeah. If you were a horse, they'd put you down (laughter). Just joking me, but obviously, it has affected me in the way that I've had to go out of work to go over to get physio. And I have had this time off, so I don't know if they're a bit, 'Well, it's not that bad.' Because day-to-day I try to be as normal as I can." [P9].

Loss of significant relationships has emerged as a key aspect of loss in previous studies of patients with persistent pain;[34–36] and disruption to important and meaningful relationships was a strong and common theme found in patients with PFP. For example:

"I've missed out of things over the years, spending time with friends, spending time with family and that kind of thing, because I've not been able to do it." [P6].

As identified by the above extracts, PFP had a compelling and far reaching impact on the participants and their lives. The pain and its disruption to life; loss of self-identify; and loss of relationships were themes that emerged from the data.

Theme 2: uncertainty, confusion and sense making

Confusion and sense making formed a central part in the lives of the participants, with a strong desire from all to elucidate the cause of their pain.

For peer review only

"If I could find out what it was that was causing the pain, then you hope it would be gone within a year. But because we don't really know what's caused it, it's kinda trial and error. So I don't really know." [P1].

The predominant focus of the participants' beliefs and attempts at making sense of their pain was that biomechanical factors were causative, with individuals trying to link these factors to the development and maintenance of their pain.

"My running technique or, I'm not sure. I'm not sure about that. I'm not sure. I think that's one thing, maybe something to do with the running technique, or something, or something to do with that." [P8].

Furthermore, confusion was also related to the episodic nature of the symptoms, with participants attempting to relate 'flare-ups' to the same biomedical factors.

A number of participants told stories of structural and biomedical beliefs becoming deep-rooted and established when reinforced. For example, one participant recounted multiple encounters with healthcare practitioners that influenced and reinforced her structural belief.

"The work physio guy said to me that he thinks that my heels have maybe gone in which has then pulled my kneecap out of alignment. So instead of going smoothly over the joint where it's supposed to, that it's probably moving over the bone and that's the sharp pain that I'm feeling. Which did make sense because it, like I said, felt like I'd got a rock underneath my kneecap at some stage." [P9].

Some participants remembered biomechanical focused diagnoses they had been given by a healthcare practitioner they had seen many years in the past; highlighting the power and lasting influence healthcare practitioners have on their patients. For example one participant remembered the diagnosis she had received from a healthcare practitioner over 10 years ago:

"I had to go to the hospital once to have x-rays… I don't know if he [doctor] was trying to scare me into doing some exercise or something, but he basically said the only thing they could do is break both of my thighs and twist them a bit and then heal them back together. And it would take me years to get back to walking properly." [P4].

Joint noises are a common feature of normal joint movement,[22] however participants commonly reported distress and confusion at joint noises, often finding healthcare practitioners' explanations inadequate.

"It was the noise that was concerning me more than the pain. I'm used to hurting. I'm too small to play rugby for a start, and I'd been fighting for 20 years, so, erm, it's one of those, you get used to the pain, but it's just the noise. When you start, you sort of [say] no, that's not right." [P3].

This was in agreement with previous research, which identified negative emotions and inaccurate etiological beliefs with joint noises in patients with PFP.[37]

For peer review only

Expressly linked to participants' confusion and need to find the cause of their pain was also a strong desire to pursue radiological imaging, and feelings of not being taken fully seriously by the healthcare profession when this was not forthcoming.

> *"I want to know exactly what the problem is. Obviously, the doctor said, previously going back, they said tendonitis, and now they're saying it's runner's knee or whatever. But you know, it's still like, is that 100%, are you sure that's what it is? Because I was going to ask the doctor to send me for a MRI..." [P8].*

Previous research has linked poor outcomes with radiological imaging in populations with low back pain, suggesting an over use of imaging has a detrimental effect on outcomes.[38] There was one example of the resulting radiological findings compounding the confusion and distrust, for example Participant Six explained her feelings on a normal MRI finding as:

> *"I mean I was a bit concerned, because they didn't turn around and say, you have hurt it, but it's not major but this is what you've done, but they didn't actually, they said nothing's wrong, take the knee brace off, and carry on. [I was] almost deflated, because I was like wanting to know why it was hurting, but they weren't explaining any of that to me. So it's a bit like, difficult." [P6].*

Another participant's story demonstrates the negative impact of discordance between healthcare practitioners' diagnosis and advice, further compounding confusion and mistrust:

> *"Well, it makes you wonder then which one to believe, because I'm like, 'Well okay, he's told me not to do anything until I'm pain-free, because he doesn't want me to aggravate it,' but when, when I came here, and obviously they said that it would probably be best to start putting an impact on it again … " [P9].*

The sense-making processes that participants described were established from past experience of healthcare treatment, past experience of pain and cultural beliefs around structure and pain.

Theme 3: exercise and activity beliefs

All participants identified specific beliefs regarding barriers to exercise and activity. These were informed by factors relating to: diagnosis uncertainty; cultural beliefs around pain; fear-avoidant behaviours and the iatrogenic effect of healthcare.

Diagnosis uncertainty, contributed to participants' beliefs regarding exercise and activity. In particular, it underpinned a dilemma regarding the relationship between activity and potential harm:

> *"It's 'are you making it worse?' And that's the crux of it really. As I'm doing it and thinking, 'if this is hurting, should I really be doing this, or shall I pack this in and do something else?' But it's the not knowing …" [P5].*

Cultural beliefs around pain being a direct sign of tissue damage was evident in a large proportion of the participants' narratives, resulting in negative behaviour towards exercise and activity.

> *"…with me it's always been, if something hurt it because your body's telling you if you do that you're going to cause more injury. You'll make things worse." [P6].*

For peer review only

Associated with the cultural beliefs on pain and damage was the resultant fear-avoidant behaviour. Participants, frequently contradicted themselves however; many participants would express the sentiment that they would not let the pain stop them from doing what they wanted to do, yet demonstrated clear activity withdrawal.

> *"So for example, we went to [holiday resort] last year; on your feet all day, walking miles and miles, I would be, like, in tears by the end of the day. I wouldn't let it stop me the next day because I would be, like, I'm doing this" [P4].*

> *"When I was in [holiday resort]; a couple of days I didn't go out and I stayed back at the hotel. Because I couldn't do it, I needed to rest." [P4].*

A predominant sub-theme was the association of sport and exercise, even in the absence of pain, as a potential precursor to future joint pain and 'damage'. Some participants attributed their current PFP to past sporting activities, despite no obvious mechanisms of injury.

> *"Yeah. Obviously it stems from doing long distance running." [P7].*

A number of participants discussed the direct impact of healthcare practitioner's advice and diagnosis labelling on their exercise and activity levels, suggesting an iatrogenic effect of healthcare for PFP patients.

> *"I have been told by doctors before I shouldn't run because it would jar my knee and shouldn't run or walk on an uneven surface because it will wonk my knee from side to side." [P4].*

> *"But then when I started the physio at work and he told me that I shouldn't walk or that I shouldn't swim because he just wanted to obviously manipulate it and get me pain-free before I did anything that could possibly aggravate it. So I stopped." [P9].*

This theme identified a number of beliefs associated as a barrier to activity and exercise engagement. These included diagnosis uncertainty; cultural beliefs around pain; fear-avoidant behaviours and the iatrogenic effect of healthcare.

Theme 4: behavioural coping strategies

A central coping strategy for participants of this study was the concept of rest. Many of them associated rest, and avoidance of activity, with the idea that time was necessary for the healing process, and that aggravating activities should be avoided.

> *"I try, obviously, sit down as much as I can." [P4].*

One participant expressed an expectation that healthcare professionals would advise him not to continue with activity and exercise:

> ***R:*** *So you think physios would say no [to keep physically active]?*

For peer review only - http://bmjopen.bmj.com/site/about/guidelines.xhtml

***P8:** Physios would probably say no. Yeah, you shouldn't do it.*

Another common coping strategy was postural adjustments; participants often talked of preferred sitting positions in relation to knee flexion.

In keeping with previous research on the high levels of analgesic use in patients with PFP,[7] a common narrative shared with participants was the use of analgesics, with some acknowledging they were not effective.

> *"I have had some strong painkillers from the doctors. They gave me some naproxen and some codeine to manage it when it was at its worst but I try not to take them." [P9].*

The use of knee supports was also common in the self-management strategies employed by the participants.

> *"If it hurts, it hurts. I'll try and strap my knee up. Because if I know I'm going harder in like gym classes, I'll strap my knees up before I go. And then when I get too much pain, I'll stop the exercise." [P10].*

Theme 5: expectations of the future

A number of participants expressed views, which could be contextualised as an external locus of control, with expectations of passive physiotherapeutic treatment options.

> *"I would presume manipulation of muscles groups, joints and tendons." [P3].*

Even though the majority of participants expressed negative views about the future, they all expressed a desire to be pain free, over and above any functional improvements.

***R:** With the physio, what would you class as a success?*

***P8:** Getting rid of the pain.*

Nine of the ten participants held negative beliefs about the future; particularly in relation to prognostic prediction following their referral to physiotherapy.

> *"But then when I'm going up the stairs and it hurts it does concern me that it's going to be every day for the rest of my life I'm going to be struggling to walk upstairs. And then I think about getting old, and I think I'm going to end up with a stair lift and living downstairs and that sort of thing." [P1].*

> *" [the pain is] definitely preying on my mind. Is it gonna stop me from going into the police, is that gonna stop me doing the things I want to do later on in life? So yeah, it does prey on my mind a little bit." [P6].*

Central to their negative beliefs about the future and their prognosis was low self-efficacy. Participants felt they had very little control over their symptoms.

For peer review only

"[In] my head, my thought process is I just hate it. Do an operation. Get rid of it. In my head, and obviously not being from the medical profession, but I'm just like, "Just get rid of the pain however it can be done." [P8].

"Yes, I'm 37 now and they feel older than that. You just get that feeling, don't you, I've bounced back from lots of injuries before but this is the one that is making me think. You know, when this gets cold I can feel it, and thinking there's already arthritis there, I'm in trouble, it sets the brain going." [P3].

Low expectation of physiotherapy, and past physiotherapy failed treatments were also a core theme within future expectations.

R: *Have you got any expectations of what might happen when you walk in to see the physio?*

P10: *I expect them to turn around and say physio can't help.*

"When I did get the physiotherapy it kinda didn't really do anything anyway. So it just made me think, it's pointless, 'cause they was trying to remove the fluid from out my knee, that like I say, made it worse to begin with. She did say your knees will feel sore, but it went back to how it was anyway, so, it just seemed like a pointless process." [P7].

There was one exception, with one participant having positive outlook to the future and their physiotherapy referral.

"Oh yeah, I think it will get better. Yeah, I'd go for the better option." [P9].

The main sub-themes that emerged under the future were: beliefs that their pain will get worse; external locus of control with regards to treatment; low self-efficacy; poor opinion of physiotherapy and previous failed physiotherapy treatments and an overwhelming desire to be pain free, over and above any practical goals for rehabilitation.

Discussion

Main Findings

Quantitative research methodologies dominate the literature for PFP. This is the first study to use a qualitative method of inquiry to gain data on the experiences of people living with PFP. The five major themes that emerged from the data were: (1) impact on self; (2) uncertainty, confusion and sense making; (3) exercise and activity beliefs; (4) behavioural coping strategies and (5) expectations of the future.

A key finding of this study is that loss of physical ability is profound and considerable, and plays a significant role in participants' lives; despite previous research suggesting that PFP is a benign and

For peer review only

self-limiting condition.[5] An inability to continue with significant and meaningful activities has been identified as a cause of anxiety in people with persistent pain.[39] Persistent pain interrupts behaviour and a person's self-identity by affecting a sense of who they are, and what they might become.[40] As a result, lives are socially and environmentally restricted by persistent interruptions, or an inability to complete, or even attempt important tasks and activities.[40] With changes and loss of participants' position and role, for example with employment or family duties, the internalised meanings and expectations associated with one's self-identity is further threatened.[32]

Participants expressed intense confusion around their pain and symptoms. For instance, the causative reasons were elusive and troubling, as too was the ability to predict and control the pain intensity; and any attempts that participants made at understanding were firmly within the biomechanical sphere of reasoning. An inability to make sense of pain, and the process associated with sense-making and pain-related fear has been proposed in low back pain populations.[41] Previous research has identified that an inability to make sense of pain places 'lives on hold',[42] and may lead to more 'catastrophising'.[43]

There remains scientific debate and uncertainty around the underlying aetiology of PFP,[44] and there is a large variation in the way PFP is managed by physiotherapists in the UK.[45] The majority of participants in this study had previous experience of healthcare management for PFP suggesting that variation in healthcare treatment may have a negative impact on the patients' lived experience. Historically the biomedical model of pain establishes a direct relationship between tissue structure and pain,[46] and participants characteristically attributed their pain to structure and/or anatomical problems. However several studies have recently demonstrated that structural abnormalities of the patellofemoral joint on Magnetic Resonance Imaging (MRI) are not associated with PFP.[47,48] Three participants had no previous healthcare management for PFP, but nevertheless gave a biomechanical/structural cause for their pain; all three had previous physiotherapy for other pain conditions, including back, hips and ankles. This may suggest that exposure to biomechanical approaches to the management of musculoskeletal pain in general could, potentially, have a carryover to other locations of pain, with a negative effect.

The iatrogenic effect of healthcare is an emerging field of research in the low back pain population [38,49]. This study is the first to find such a theme in patients with PFP. These findings are consistent with recent research that showed that the majority of UK physiotherapists would advise their patients not to continue with exercises if they experienced any pain.[45] The fear-avoidance model of pain is a well-established with patients with persistent pain, particularly persistent low back pain;[17] additionally research has shown that fear-avoidance behaviour may also exist with clinicians.[25,45,50] The central concept of the model is cognitions and emotions that underpin fear of the pain; fears about potential physical activities exacerbating the pain and further 'damaging' bodies. The fear leads to safety seeking behaviours and hypervigilance that paradoxically maintains or exacerbates the pain and disability.[22] In contrast, if pain is perceived in a non-threatening way patients are likely to maintain physical activity levels, through which recovery can be achieved.[51,52] All of the ten participants in this study described fear-avoidant behaviour at some stage of their interview. This is the first study, which we know of, that identifies this behaviour in patients with a diagnosis of PFP.

Patellofemoral pain is often described as an 'overuse' injury,[53] and these data seem to be consistent with the patients' belief and behaviour with a definition more aligned with the English language meaning of 'overuse'. Contemporary thinking in relation to injury risk challenges the idea that PFP is

simply an 'overuse' injury, with evidence suggesting that persistent and long-term under-use may be a risk factor, with consistent exposure to tissue load being considered one method of management.[54] The fear-avoidant behaviours revealed within this study would therefore be seen as negative pain behaviour, with long-term detrimental consequences.

A key finding of this research is the low expectation for the future and low self-efficacy demonstrated by the majority of the participants that could be conceptualised as 'catastrophising'. Catastrophising is conceptually within the same model of pain behaviour as fear-avoidance, with largescale overlap.[19] Low self-efficacy, fear of the future and catastrophising is a common finding in patients with persistent pain.[24,55] The National Institute of Health and Care Excellence describes pain as a complex biopsychosocial issue, associated with expectations, self-efficacy, mood and coping abilities.[56] In addition, it has been shown that self-efficacy is a strong predictor of successful outcome, irrespective of the intervention delivered, for patients with persistent pain; suggesting that rehabilitation programmes for persistent musculoskeletal pain should be designed with the aim of improving self-efficacy.[57]

Clinical and research implications

This study established that a sample of patients with PFP demonstrated: pain-related fear, such as fear-avoidance; damage beliefs; difficulty with making sense of their pain; low self-efficacy and fear of the future.

The current consensus that best evidence treatments consisting of hip and knee strengthening may not be adequate to address the fears and beliefs identified in the current study. Future studies are needed to explore biopsychosocial targeted interventions for this population, particularly in relation to pain experienced by patients during exercise, followed by efficacy and effectiveness trials. Interventions may be patient education packages and self-management strategies targeting self-efficacy and physical activity. Furthermore, future qualitative work will be beneficial to understand the role of medical terminology commonly used with this patient group, for example, 'weakness' and 'patellar mal-tracking',[45] and its impact and interpretation by patients.

Study limitations and strengths

Two authors independently coded all transcripts, and this study employed a clear, transparent and reproducible methodological approach to data analysis. The authors make it clear that their clinical and research experience lie within the biopsychosocial framework of musculoskeletal pain and this study forms part of a larger body of research looking at pain education, self-management strategies and exercise interventions for individuals with PFP.[58] It is worth noting that the interviewer made it explicit to the participants that he was a physiotherapist working in the department conducting the research; indeed a number of them did proceed to ask clinical questions about their condition, highlighting a power dynamic between the interviewer and participant. Furthermore, it is important to note that recruitment took place in the same department that the researcher was working as a physiotherapist. This may, is part, have influenced participants' inclination to take part, and also their responses.

The main limitation of this study is that for pragmatic reasons a convenience sampling technique was used. It is possible that this sample may differ from other samples within the UK, and how representative these findings are to the greater population of individuals with PFP is unknown. A

For peer review only

purposive sampling technique may have better represented sociodemographic groups, or targeted identifiable subgroups.

Conclusion

These findings offer an insight into the experience of individuals living with PFP. Previous literature have focused on pain and biomechanics, rather than the individual experience, attached meanings and any wider context within a sociocultural perspective. The participants provided rich and detailed narratives of loss of physical and functional ability; loss of self-identity; pain related confusion and difficulty making sense of their pain; pain-related fear, including fear-avoidance and 'damage' beliefs; inappropriate coping strategies and fear of the future. Our findings suggest future research is warranted into biopsychosocial targeted interventions and the impact and interpretation of medical terminology.

Authors' contributions

BES was responsible for conception and design, compiling the interview schedule, interviewing, transcribing, coding, analysis and interpretation, drafting and revising the manuscript. FM was responsible for conception and design, compiling the interview schedule, coding, analysis and interpretation, drafting and revising the manuscript. PH, MB, JS, MR, TS and PL were involved in conception and design, interpretation and reviewing revisions to the manuscript. All authors have read and approved of the final manuscript.

Funding

This report is independent research arising from a Clinical Doctoral Research Fellowship, Benjamin Smith, ICA-CDRF-2015-01-002 supported by the National Institute for Health Research (NIHR) and Health Education England (HEE). The views expressed in this publication are those of the author(s) and not necessarily those of the NHS, the NIHR, HEE or the Department of Health.

Competing interests

The authors declare that they have no competing interests.

Ethics approval

This study was approved by the West Midlands - Black Country Research Ethics Committee (16/WM/0414).

Availability of data

Quotations and further details are available from Benjamin Smith at benjamin.smith3@nhs.net

Supplementary Data

Data supplement 1 – Code Book

For peer review only

References

1 Dey P, Callaghan M, Cook N, *et al.* A questionnaire to identify patellofemoral pain in the community: an exploration of measurement properties. *BMC Musculoskelet Disord* 2016;17:237. doi:10.1186/s12891-016-1097-5

2 Urwin M, Symmons D, Allison T, *et al.* Estimating the burden of musculoskeletal disorders in the community: the comparative prevalence of symptoms at diVerent anatomical sites, and the relation to social deprivation. *Ann Rheum Dis* 1998;57:649–55.

3 Jordan KP, Kadam UT, Hayward R, *et al.* Annual consultation prevalence of regional musculoskeletal problems in primary care: an observational study. *BMC Musculoskelet Disord* 2010;11:144. doi:10.1186/1471-2474-11-144

4 Crossley KM, Stefanik JJ, Selfe J, *et al.* 2016 Patellofemoral pain consensus statement from the 4th International Patellofemoral Pain Research Retreat, Manchester. Part 1: Terminology, definitions, clinical examination, natural history, patellofemoral osteoarthritis and patient-reported outcome m. *Br J Sports Med* 2016;50:839–43.

5 Sandow MJ, Goodfellow JW. The natural history of anterior knee pain in adolescents. *J Bone Joint Surg Br* 1985;67:36–8.

6 Collins NJ, Bierma-Zeinstra SMA, Crossley KM, *et al.* Prognostic factors for patellofemoral pain: a multicentre observational analysis. *Br J Sports Med* 2013;47:227–33. doi:10.1136/bjsports-2012-091696

7 Stathopulu E, Baildam E. Anterior knee pain: a long-term follow-up. *Rheumatology (Oxford)* 2003;42:380–2.

8 Rathleff MS, Rasmussen S, Olesen JL. Unsatisfactory long-term prognosis of conservative treatment of patellofemoral pain syndrome. *Ugeskr Laeger* 2012;174:1008–13.

9 Smith BE, Hendrick P, Logan P. Patellofemoral pain: Challenging current practice - A case report. *Man Ther* 2016;22:216–9. doi:10.1016/j.math.2015.09.002

10 Doménech J, Sanchis-Alfonso V, López L, *et al.* Influence of kinesiophobia and catastrophizing on pain and disability in anterior knee pain patients. *Knee Surgery, Sport Traumatol Arthrosc* 2013;21:1562–8. doi:10.1007/s00167-012-2238-5

11 Piva SR, Fitzgerald GK, Wisniewski S, *et al.* Predictors of pain and function outcome after rehabilitation in patients with patellofemoral pain syndrome. *J Rehabil Med* 2009;41:604–12. doi:10.2340/16501977-0372

For peer review only

12 Piva SR, Fitzgerald GK, Irrgang JJ, *et al.* Associates of Physical Function and Pain in Patients with Patellofemoral Pain Syndrome. *Arch Phys Med Rehabil* 2009;90:285–95. doi:10.1016/j.apmr.2008.08.214

13 Quartana PJ, Campbell CM, Edwards RR. Pain catastrophizing: a critical review. *Expert Rev Neurother* 2009;9:745–58. doi:10.1586/ern.09.34

14 Moseley GL, Arntz A. The context of a noxious stimulus affects the pain it evokes. *PAIN®* 2007;133:64–71.

15 Harvie DS, Broecker M, Smith RT, *et al.* Bogus visual feedback alters onset of movement-evoked pain in people with neck pain. *Psychol Sci* 2015;:0956797614563339.

16 Lobanov O V, Zeidan F, McHaffie JG, *et al.* From cue to meaning: Brain mechanisms supporting the construction of expectations of pain. *PAIN®* 2014;155:129–36.

17 Chou R, Shekelle P. Will this patient develop persistent disabling low back pain? *JAMA* 2010;303:1295–302. doi:10.1001/jama.2010.344

18 Mallows A, Debenham J, Walker T, *et al.* Association of psychological variables and outcome in tendinopathy: a systematic review. *Br J Sports Med* 2016;:bjsports – 2016.

19 Wertli MM, Rasmussen-Barr E, Weiser S, *et al.* The role of fear avoidance beliefs as a prognostic factor for outcome in patients with nonspecific low back pain: a systematic review. *spine J* 2014;14:816–36.

20 Osborn M, Smith JA. The personal experience of chronic benign lower back pain: An interpretative phenomenological analysis. *Br J Pain* 2015;9:65–83.

21 Smith JA, Osborn M. Pain as an assault on the self: An interpretative phenomenological analysis of the psychological impact of chronic benign low back pain. *Psychol Heal* 2007;22:517–34.

22 Jones S, Hanchard N, Hamilton S, *et al.* A qualitative study of patients’ perceptions and priorities when living with primary frozen shoulder. *BMJ Open* 2013;3:e003452.

23 Walker J, Holloway I, Sofaer B. In the system: the lived experience of chronic back pain from the perspectives of those seeking help from pain clinics. *Pain* 1999;80:621–8.

24 Walker J, Sofaer B, Holloway I. The experience of chronic back pain: accounts of loss in those seeking help from pain clinics. *Eur J Pain* 2006;10:199.

For peer review only

25 Nijs J, Roussel N, Paul van Wilgen C, *et al.* Thinking beyond muscles and joints: Therapists' and patients' attitudes and beliefs regarding chronic musculoskeletal pain are key to applying effective treatment. *Man Ther* 2013;18:96–102.

26 Thorne S, Kirkham SR, MacDonald-Emes J. Focus on qualitative methods. Interpretive description: a noncategorical qualitative alternative for developing nursing knowledge. *Res Nurs Health* 1997;20:169–77.

27 Braun V, Clarke V. Using thematic analysis in psychology. *Qual Res Psychol* 2006;3:77–101.

28 Tong A, Sainsbury P, Craig J. Consolidated criteria for reporting qualitative research (COREQ): a 32-item checklist for interviews and focus groups. *Int J Qual Heal care* 2007;19:349–57.

29 Mc Auliffe S, Synott A, Casey H, *et al.* Beyond the tendon: Experiences and perceptions of people with persistent Achilles tendinopathy. *Musculoskelet Sci Pract* 2017;29:108–14.

30 Leary MR, Tangney JP. *Handbook of self and identity*. Guilford Press 2011.

31 Baumeister RF. *The self in social psychology*. 1999.

32 Stryker S, Burke PJ. The past, present, and future of an identity theory. *Soc Psychol Q* 2000;:284–97.

33 Lamé IE, Peters ML, Vlaeyen JWS, *et al.* Quality of life in chronic pain is more associated with beliefs about pain, than with pain intensity. *Eur J Pain* 2005;9:15–24.

34 Aldrich S, Eccleston C. Making sense of everyday pain. *Soc Sci Med* 2000;50:1631–41.

35 Charmaz K. From the' sick role' to stories of self: Understanding the self in illness. *Rutgers Ser Self Soc Identity* 1999;2:209–39.

36 Johansson EE, Hamberg K, Westman G, *et al.* The meanings of pain: an exploration of women's descriptions of symptoms. *Soc Sci Med* 1999;48:1791–802.

37 Robertson CJ, Hurley M, Jones F. People's beliefs about the meaning of crepitus in patellofemoral pain and the impact of these beliefs on their behaviour: A qualitative study. *Musculoskelet Sci Pract* 2017;28:59–64. doi:10.1016/j.msksp.2017.01.012

38 Webster BS, Bauer AZ, Choi Y, *et al.* Iatrogenic consequences of early magnetic resonance imaging in acute, work-related, disabling low back pain. *Spine (Phila Pa 1976)* 2013;38:1939–46.

For peer review only

39 Roy R. *Chronic pain, loss and suffering*. University of Toronto Press 2004.

40 Harris S, Morley S, Barton SB. Role loss and emotional adjustment in chronic pain. *Pain* 2003;105:363–70.

41 Bunzli S, Smith A, Schütze R, *et al.* Beliefs underlying pain-related fear and how they evolve: a qualitative investigation in people with chronic back pain and high pain-related fear. *BMJ Open* 2015;5.

42 Bunzli S, Watkins R, Smith A, *et al.* Lives on hold: a qualitative synthesis exploring the experience of chronic low-back pain. *Clin J Pain* 2013;29:907–16.

43 van Wilgen CP, van Ittersum MW, Kaptein AA, *et al.* Illness perceptions in patients with fibromyalgia and their relationship to quality of life and catastrophizing. *Arthritis Rheum* 2008;58:3618–26.

44 Doménech J, Sanchis-Alfonso V, Espejo BBB, *et al.* Changes in catastrophizing and kinesiophobia are predictive of changes in disability and pain after treatment in patients with anterior knee pain. *Knee Surg Sports Traumatol Arthrosc* 2014;22:2295–300. doi:10.1007/s00167-014-2968-7

45 Smith BE, Hendrick P, Bateman M, *et al.* Current Management Strategies for Patellofemoral Pain: An online survey of 99 practising UK physiotherapists. *BioMed Cent Musculoskelet Disord* 2017;18. doi:10.1186/s12891-017-1539-8

46 Doménech J, Sanchis-Alfonso V, Espejo B. Influence of Psychological Factors on Pain and Disability in Anterior Knee Pain Patients. In: *Anterior Knee Pain and Patellar Instability*. London: : Springer London 2011. 123–35. doi:10.1007/978-0-85729-507-1_9

47 van der Heijden RA, Oei EHG, Bron EE, *et al.* No Difference on Quantitative Magnetic Resonance Imaging in Patellofemoral Cartilage Composition Between Patients With Patellofemoral Pain and Healthy Controls. *Am J Sports Med* 2016;44:1172–8. doi:10.1177/0363546516632507

48 van der Heijden RA, de Kanter JLM, Bierma-Zeinstra SMA, *et al.* Structural Abnormalities on Magnetic Resonance Imaging in Patients With Patellofemoral Pain: A Cross-sectional Case-Control Study. *Am J Sports Med* 2016;44:2339–46. doi:10.1177/0363546516646107

49 Lin IB, O'Sullivan PB, Coffin JA, *et al.* Disabling chronic low back pain as an iatrogenic disorder: a qualitative study in Aboriginal Australians. *BMJ Open* 2013;3:1–8.

50 Littlewood C, Lowe A, Moore J. Rotator cuff disorders: a survey of current UK physiotherapy practice. *Shoulder Elb* 2012;4:64–71. doi:10.1111/j.1758-5740.2011.00164.x

For peer review only

51 Leeuw M, Goossens MEJB, Linton SJ, *et al.* The fear-avoidance model of musculoskeletal pain: current state of scientific evidence. *J Behav Med* 2007;30:77–94. doi:10.1007/s10865-006-9085-0

52 Vlaeyen JW, Linton SJ. Fear-avoidance and its consequences in chronic musculoskeletal pain: a state of the art. *Pain* 2000;85:317–32.

53 Herrington L, Nester C. Q-angle undervalued? The relationship between Q-angle and medio-lateral position of the patella. *Clin Biomech (Bristol, Avon)* 2004;19:1070–3. doi:10.1016/j.clinbiomech.2004.07.010

54 Gabbett TJ. The training-injury prevention paradox: should athletes be training smarter and harder? *Br J Sports Med* 2016;50:273–80. doi:10.1136/bjsports-2015-095788

55 Arnstein P, Caudill M, Mandle CL, *et al.* Self efficacy as a mediator of the relationship between pain intensity, disability and depression in chronic pain patients. *Pain* 1999;80:483–91.

56 National Institute for Clinical Excellence. Osteoarthritis: care and management. 2014.

57 Miles CL, Pincus T, Carnes D, *et al.* Can we identify how programmes aimed at promoting self-management in musculoskeletal pain work and who benefits? A systematic review of sub-group analysis within RCTs. *Eur J Pain* 2011;15. doi:10.1016/j.ejpain.2011.01.016

58 Smith BE, Hendrick P, Bateman M, *et al.* Study protocol: a mixed methods feasibility study for a loaded self-managed exercise programme for patellofemoral pain. *Pilot Feasibility Stud* 2017;4. doi:10.1186/s40814-017-0167-2

For peer review only

Data supplement 1 – Code Book

Name

Impact on self, sense of loss

Name
Pain experiences, disruption to life, distressing
Loss of identity, self
Relationships loss (time with family, friends, children, friends making fun, judgement)
Knee noises experiences, distressing

Not understanding, sense making, confusion

Name
Trying to find cause
Anatomy and imaging central to causation
Historical diagnosis – i.e historical comments still central to beliefs
Distrust of healthcare – wanting scans
Not being taken seriously
Disagreement with healthcare increases uncertainty and distress

Exercise and Activity Barriers

Name
Diagnosis uncertainty
Cultural beliefs around pain
Fear avoidance
Iatrogenic effect of healthcare
Sport = future 'damage'
Physio = exercise
contradiction

Coping Strategies

Name
Central to coping is activity avoidance – despite saying they don't let it stop them
Postural adjustments
Rest
Analgesics – whilst acknowledging they don't work
Reliance on knee support – not knowing how they work

The Future

Name
Belief will get worse
Effect of healthcare on that belief (including external locus of control)
Low self-efficacy
Low opinion of physio, past physio failures
Desire to be pain free
high expectation of prognosis

For peer review only

Consolidated criteria for reporting qualitative studies (COREQ): 32-item checklist

Developed from:
Tong A, Sainsbury P, Craig J. Consolidated criteria for reporting qualitative research (COREQ): a 32-item checklist for interviews and focus groups. *International Journal for Quality in Health Care*. 2007. Volume 19, Number 6: pp. 349 – 357

YOU MUST PROVIDE A RESPONSE FOR ALL ITEMS. ENTER N/A IF NOT APPLICABLE

No. Item	Guide questions/description	Reported on Page #
**Domain 1: Research team and reflexivity**		
Personal Characteristics		
1. Inter viewer/facilitator	Which author/s conducted the inter view or focus group?	Page 5
2. Credentials	What were the researcher's credentials? E.g. PhD, MD	Page 5
3. Occupation	What was their occupation at the time of the study?	Page 5
4. Gender	Was the researcher male or female?	Page 5
5. Experience and training	What experience or training did the researcher have?	Page 5
Relationship with participants		
6. Relationship established	Was a relationship established prior to study commencement?	Page 5
7. Participant knowledge of the interviewer	What did the participants know about the researcher? e.g. personal goals, reasons for doing the research	Page 5
8. Interviewer characteristics	What characteristics were reported about the inter viewer/facilitator? e.g. Bias, assumptions, reasons and interests in the research topic	Page 5 & 15
**Domain 2: study design**		
Theoretical framework		
9. Methodological orientation and Theory	What methodological orientation was stated to underpin the study? e.g. grounded theory, discourse analysis, ethnography, phenomenology, content analysis	Page 5
Participant selection		
10. Sampling	How were participants selected? e.g. purposive, convenience, consecutive, snowball	Page 5
11. Method of approach	How were participants approached? e.g. face-to-face, telephone, mail, email	Page 5
12. Sample size	How many participants were in the study?	Page 5

For peer review only

13. Non-participation	How many people refused to participate or dropped out? Reasons?	Page 5
Setting		
14. Setting of data collection	Where was the data collected? e.g. home, clinic, workplace	Page 5
15. Presence of non-participants	Was anyone else present besides the participants and researchers?	Page 5
16. Description of sample	What are the important characteristics of the sample? e.g. demographic data, date	Page 7
Data collection		
17. Interview guide	Were questions, prompts, guides provided by the authors? Was it pilot tested?	Page 5
18. Repeat interviews	Were repeat inter views carried out? If yes, how many?	N/A
19. Audio/visual recording	Did the research use audio or visual recording to collect the data?	Page 5
20. Field notes	Were field notes made during and/or after the inter view or focus group?	Page 5 & 6
21. Duration	What was the duration of the inter views or focus group?	Page 7
22. Data saturation	Was data saturation discussed?	Page 6
23. Transcripts returned	Were transcripts returned to participants for comment and/or correction?	N/A
**Domain 3: analysis and findings**		
Data analysis		
24. Number of data coders	How many data coders coded the data?	Page 6
25. Description of the coding tree	Did authors provide a description of the coding tree?	N/A
26. Derivation of themes	Were themes identified in advance or derived from the data?	Page 6
27. Software	What software, if applicable, was used to manage the data?	NVivo
28. Participant checking	Did participants provide feedback on the findings?	NO
Reporting		
29. Quotations presented	Were participant quotations presented to illustrate the themes/findings? Was each quotation identified? e.g. participant number	Results
30. Data and findings consistent	Was there consistency between the data presented and the findings?	Page 13
31. Clarity of major themes	Were major themes clearly presented in the findings?	RESULTS
32. Clarity of minor themes	Is there a description of diverse cases or discussion of minor themes?	Discussion

Once you have completed this checklist, please save a copy and upload it as part of your submission. When requested to do so as part of the upload process, please select the file type: *Checklist*. You will NOT be able to proceed with

For peer review only

submission unless the checklist has been uploaded. Please DO NOT include this checklist as part of the main manuscript document. It must be uploaded as a separate file.

For peer review only

BMJ Open

The experience of living with patellofemoral pain: loss, confusion and fear-avoidance – a UK qualitative study

Journal:	*BMJ Open*
Manuscript ID	bmjopen-2017-018624.R3
Article Type:	Research
Date Submitted by the Author:	30-Nov-2017
Complete List of Authors:	Smith, Benjamin; Derby Teaching Hospitals NHS Foundation Trust, Physiotherapy Outpatients; University of Nottingham, Division of Rehabilitation and Ageing, School of Medicine<br>Moffatt, Fiona; University of Nottingham, Division of Physiotherapy and Rehabilitation Sciences, School of Health Sciences,<br>Hendrick, Paul ; University of Nottingham, Division of Physiotherapy and Rehabilitation Sciences, School of Health Sciences<br>Bateman, Marcus; Derby Teaching Hospitals NHS Foundation Trust, Physiotherapy Outpatients<br>Rathleff, Michael ; Aalborg University, Research Unit for General Practice in Aalborg, Department of Clinical Medicine ; Aalborg University Hospital, Department of Occupational Therapy and Physiotherapy, Department of Clinical Medicine<br>Selfe, James; Manchester Metropolitan University, Department of Health Professions<br>Smith, Toby O.; University of East Anglia, Faculty of Medicine and Health Sciences<br>Logan, Phillipa; University of Nottingham , Division of Rehabilitation and Ageing, School of Medicine
**Primary Subject Heading**:	Qualitative research
Secondary Subject Heading:	Rehabilitation medicine, Sports and exercise medicine
Keywords:	patellofemoral pain, anterior knee pain, PFP

SCHOLARONE™
Manuscripts

The experience of living with patellofemoral pain: loss, confusion and fear-avoidance – a UK qualitative study

Benjamin E Smith[1,2,*], Fiona Moffatt[3], Paul Hendrick[3], Marcus Bateman[1], Michael Skovdal Rathleff[4,5], James Selfe[6], Toby O Smith[7], Pip Logan[2]

[1]Derby Teaching Hospitals NHS Foundation Trust, Physiotherapy Department (Level 3), London Road Community Hospital, Derby DE1 2QY, UK

[2]Division of Rehabilitation and Ageing, School of Medicine, University of Nottingham, Nottingham, UK

[3]Division of Physiotherapy and Rehabilitation Sciences, School of Health Sciences, University of Nottingham, Nottingham University Hospitals (City Campus), Nottingham, UK.

[4]Research Unit for General Practice in Aalborg, Department of Clinical Medicine at Aalborg University, Aalborg, Denmark

[5]Department of Occupational Therapy and Physiotherapy, Department of Clinical Medicine, Aalborg University Hospital, Denmark

[6]Department of Health Professions, Manchester Metropolitan University, Manchester, UK

[7]Faculty of Medicine and Health Sciences, University of East Anglia, Norwich, UK

[*]Corresponding author. Tel: 01332 254631 Email: benjamin.smith3@nhs.net

For peer review only

Abstract

Objectives:

To investigate the experience of living with patellofemoral pain.

Design:

Qualitative study design using semi-structured interviews, and analysed thematically using the guidelines set out by Braun and Clarke.

Setting:

A National Health Service (NHS) physiotherapy clinic within a large UK teaching hospital.

Participants:

A convenience sample of ten participants, aged between 18 and 40, with a diagnosis of patellofemoral pain and on a physiotherapy waiting list, prior to starting physiotherapy.

Results:

Participants offered rich and detailed accounts of the impact and lived experience of patellofemoral pain, including: loss of physical and functional ability; loss of self-identity; pain related confusion and difficulty making sense of their pain; pain related fear, including fear-avoidance and 'damage' beliefs; inappropriate coping strategies and fear of the future. The five major themes that emerged from the data were: (1) impact on self; (2) uncertainty, confusion and sense making; (3) exercise and activity beliefs; (4) behavioural coping strategies and (5) expectations of the future.

Conclusions:

These findings offer an insight into the lived experience of individuals with patellofemoral pain. Previous literature has focused on pain and biomechanics, rather than the individual experience, attached meanings and any wider context within a sociocultural perspective. Our findings suggest future research is warranted into biopsychosocial targeted interventions aimed at the beliefs and pain related fear for people with patellofemoral pain. The current consensus that best-evidence treatments consisting of hip and knee strengthening may not be adequate to address the fears and beliefs identified in the current study. Further qualitative research may be warranted on the impact and interpretation of medical terminology commonly used with this patient group, for example, 'weakness' and 'patellar mal-tracking' and its impact and interpretation by patients.

Trial registration:

ISRCTN 35272486

Article Summary

Strengths and limitations of this study:

- This is the first study to use a qualitative method of inquiry on the experience of people living with patellofemoral pain.
- Two authors independently coded all transcripts, and a clear, transparent and reproducible methodological approach was used in the thematic analysis.
- For pragmatic reasons a convenience sampling technique was used.

For peer review only

Introduction

Patellofemoral pain (PFP) is one of the most common and costly forms of knee pain.[1–3] It has an estimated prevalence of 23% in the general population in the UK.[1] Symptoms typically include retro-patellar or diffuse peripatellar pain, aggravated by activities that load the joint, such as climbing and descending stairs, squatting and running.[4]

Historically PFP has been labelled a “benign, self-limiting condition”, that improves over time with little intervention indicated.[5] However, this belief has recently been challenged with data suggesting that the overall long-term prognosis for the majority of patients with PFP is poor.[6] Only one third of patients are pain-free one year after diagnosis,[6] and 91% still report pain and dysfunction four years post-diagnosis.[7] Quantitative data suggests that some patients withdraw from participation in physical activities,[8,9] and may develop associated psychological distress, such as fear-avoidance and catastrophising thoughts in relation to their knee pain.[10–12]

The biopsychosocial model of persistent pain has recognised that psychological factors, such as fear and catastrophising can, through changes to behaviour, modulate physiological responses to pain with the development and maintenance of persistent pain.[13–17] Psychological distress has been identified in low back pain and tendon pain populations through systematic reviews,[18,19] and qualitative methods in low back and shoulder populations,[20–22] however to our knowledge this has not been investigated in PFP. Advocates of qualitative research methods suggest that qualitative inquiry can disclose the experience of people with pain, and therefore be used to understand patient motivation, social engagement and provide a wealth of information about the sociocultural context to pain.[23,24] Contemporary models of persistent pain have identified the importance of thinking beyond muscles and joints,[25] and qualitative inquiry can provide an insight that may lead to development of ideas and hypothesis generation within the context of the biopsychosocial model of pain. No study using qualitative methods has been published regarding PFP. Therefore the aim of this study was to give a more detailed account of the experience of people living with PFP, seeking secondary care within the UK.

For peer review only

Method

In order to address gaps in the literature this research focused on identifying themes within the participants' experience of living with PFP. A qualitative interpretive description design was chosen as an appropriate methodological approach.[26] Thematic analysis is the most appropriate method for this type of inquiry, as codes and themes can be created inductively to capture meaning and content without prior preconceptions allowing flexibility to generate a rich and detailed account of the data.[27]

In this study, data were analysed thematically using the guidelines set out by Braun and Clarke,[27] and was reported in line with the COnsolidated criteria for REporting Qualitative research (COREQ) checklist (see supplementary file 1).[28]

Braun and Clarke[27] describe a multi-stage approach to thematic data analysis; demonstrating clear distinction of the thematic approach, whilst allowing for the inherent flexibility in the process. They reasoned that a thematic analysis can be conducted from a both realist and constructionist paradigms, although with differing outcomes. A realist approach allows theories about individual motivation and meaning to be developed, since the epistemological position is that there is a unidirectional relationship between meaning, experience and language[27]. A constructionist perspective differs, as meaning and experience are socially produced and knowledge a human and social construct; therefore theories about individual motivation and meaning are inappropriate, and theories focus instead on sociocultural contexts[27]. This study did not set out to prove or disprove a hypotheses; it set out to generate new data from which an understanding of living with PFP might be developed. The authors wanted to take an epistemological position that recognises the experience at an individual level, and any meanings attached, whilst considering the wider context within a sociocultural perspective. Sitting central on the spectrum of realism and constructivism, this position is described as "contextualist" by Braun and Clarke[27].

Recruitment

A convenience sample of ten participants with a diagnosis of PFP were recruited from an NHS physiotherapy waiting list. Based on similar studies of other musculoskeletal conditions, we anticipated this sample size would be sufficient to reach data saturation, and was agreed a priori.[22,29] Participants were initially contacted by mail and followed up by a telephone call (BES). Thirty four information sheets were sent out, and 24 potential participants were contacted by telephone; two could not make the interview before physiotherapy was due to start; five people physiotherapy had already commenced; one reported resolution of symptoms; and six declined to participate. Inclusion criteria were participants aged 18 to 40 with signs and symptoms of PFP, defined as: anterior or retro-patellar pain reported on at least two of the following activities; prolonged sitting, ascending or descending stairs, squatting, jumping and running.[4] These were pre-screened during an initial telephone conversation. Exclusion criteria included: previous knee surgery; awaiting lower limb surgery; knee ligamentous instability; history of patellar dislocation; true knee locking or giving way; reasons to suspect systemic pathology, or acute illness; pregnancy or breast feeding; patellar or iliotibial tract tendinopathy; and those not able to speak or understand English. The exclusion criteria were screened prior to consent being taken (BES).

Data Collection

For peer review only

Participants were offered interviews at their home, or in a hospital-based physiotherapy department; all opted to be interviewed at the hospital. On arrival the researcher (BES) introduced himself as a physiotherapist working in that department, and also a researcher conducting a PhD. The researcher explained the aims of the study. Written consent and verbal consent was taken to start recording.

With reference to previous literature on low back pain, shoulder pain and tendon pain,[20–22] semi-structured interviews were designed by the researchers using a topic guideline with prompts to explore participants' experience of: living with PFP; past healthcare management; their interpretation of causation of their pain; beliefs, attitudes and behaviour in relation to their pain and expectations for the future. The semi-structured interviews allowed for a flexible interview, in a two-way conversation, allowing new ideas to be developed as they were brought up.

The researcher also maintained a reflective journal, noting down initial thoughts and ideas after each interview.[22] This identified that early interviews raised issues about other (past and present) musculoskeletal pain, and specific coping strategies employed by participants for their PFP. These were therefore incorporated into subsequent interview schedules.

Data Analysis

All audio files were collected and transcribed verbatim (BES). During transcription, initial thoughts and ideas were noted in the reflective journal. Audio files were listened to several times to check for accuracy, and transcriptions were read and re-read a number of times; this initial process of data familiarisation allowed for 'data immersion' by the researchers, and generation of preliminary ideas.[27] Data coding then identified and coded pertinent features of the data giving equal priority over the whole dataset. These steps were independently conducted by two researchers (BES & FM) who met to compare codes and develop agreement on the grouping of codes into themes. The generated themes were reviewed and refined, ensuring that they explained the data in relation to the coded data, and the whole dataset. The researchers then consulted on the final two stages; themes and sub-themes were named and defined to demonstrate a clear narrative, using compelling extracts as illustrations. Consideration was given to each theme individually, but also to how they related to the dataset as a whole and other themes.[27]

Data were organised and analysed using QSR International's NVivo 11. After ten interviews, it was determined by the researchers that data saturation had occurred as no new thoughts or concepts were generated in the later interviews.

For peer review only

Results

Participants ranged from 26 to 37 years of age (mean age 30.6), with a diagnosis of PFP for a mean duration of 78 months (range: 3 months to 16 years). For participants' characteristics see table 1. The interviews ranged from 13 to 43 minutes (mean time: 27 minutes).

Table 1 Characteristics of participants

Participant Number	Gender	Age	Duration of symptoms (m)	Type of Employment
1	F	26	60	Healthcare Worker
2	M	33	60	Builder
3	M	37	8	Office worker
4	F	26	192	Healthcare Worker
5	F	34	36	Office worker
6	F	27	84	Waitress
7	F	28	120	Technician
8	M	29	36	Office worker
9	F	36	3	Office worker
10	F	30	180	Office worker

F, female; M, male; m, months

The first theme that emerged from the data, impact on self, describes the participants' sense of loss, in relation to their self and self-identity. The further themes that emerged describe how the participants deal with this loss in a climate of uncertainty, how they understand or make decisions regarding exercise/activity and pain management, and how they prognosticate for the future. Data are presented to demonstrate the range and meaning to each theme.

Theme 1: impact on self

Participants offered rich and detailed accounts of the impact and lived experience of PFP. Loss of self and loss of self-identity was evident in the stories told by many of the participants in this study. Self and self-identity are different concepts about ways in which individuals evaluate and interpret themselves; they are nested elements that are shaped by the contexts of individual's lives, with direct influence on decisions and behaviours.[30] Self, in its broader sense, can be defined as one's individuality and process of making sense of the world around them; it is a cognitive structure that defines one's sense of worth.[31] Self-identity, however, is the cognitive structure of internalised meanings and expectations associated with one's position and role within a social network.[32]

Symptoms affected all participants' daily life, with pain being a pervasive and disruptive feature of their day, with resulting loss of physical ability:

> *"I struggle at work, bending down to get bottom shelf and getting back up, I literally have to hold onto the table to pull myself up. I can't do it off just my knees." [P7].*

For peer review only

"Yeah, well, it's a pain really because I'm walking around. I'm very stiff with that leg. Going up the stairs, down the stairs at work, getting out of a chair, getting into the car." [P6].

Several participants described the negative impact of PFP on their mental well-being, with subsequent loss of self-identity:

"I would say the reason I got my horse was because I have mental health problems and so having a horse is my routine, structure, thing that I look forward to doing. The positive in my life. And having the knee problem makes that, makes that, not so effective. You can't do, what I imagined I would be able to do." [P4].

Physical activity has been identified as a key quality of life domain, and the one most affected among patients with persistent pain.[33] Loss of activities for these participants included: walking; exercise; driving; holidays; time with family and friends; playing with children; duties at work and kneeling. These loss of activities directly affected participants' role and position within their social network, triggering feelings of loss of self-identity. For example, a number of participants explained how PFP affected their work, and made them question their career aspirations:

"I would say, it makes me like wonder, if I can do the job, not at this point but maybe when I get older and older, maybe I won't be able to do it". [P4].

Judgemental attitudes from colleagues, friends or family, were described by a number of participants, with subsequent feelings of loss of self-identity, acting as moderators to low moods and feelings of premature ageing:

"They're saying that I'm a grandma. They say, 'Yeah. If you were a horse, they'd put you down (laughter). Just joking me, but obviously, it has affected me in the way that I've had to go out of work to go over to get physio. And I have had this time off, so I don't know if they're a bit, 'Well, it's not that bad.' Because day-to-day I try to be as normal as I can." [P9].

Loss of significant relationships has emerged as a key aspect of loss in previous studies of patients with persistent pain;[34–36] and disruption to important and meaningful relationships was a strong and common theme found in patients with PFP. For example:

"I've missed out of things over the years, spending time with friends, spending time with family and that kind of thing, because I've not been able to do it." [P6].

As identified by the above extracts, PFP had a compelling and far reaching impact on the participants and their lives. The pain and its disruption to life; loss of self-identify; and loss of relationships were themes that emerged from the data.

Theme 2: uncertainty, confusion and sense making

Confusion and sense making formed a central part in the lives of the participants, with a strong desire from all to elucidate the cause of their pain.

For peer review only

"If I could find out what it was that was causing the pain, then you hope it would be gone within a year. But because we don't really know what's caused it, it's kinda trial and error. So I don't really know." [P1].

The predominant focus of the participants' beliefs and attempts at making sense of their pain was that biomechanical factors were causative, with individuals trying to link these factors to the development and maintenance of their pain.

"My running technique or, I'm not sure. I'm not sure about that. I'm not sure. I think that's one thing, maybe something to do with the running technique, or something, or something to do with that." [P8].

Furthermore, confusion was also related to the episodic nature of the symptoms, with participants attempting to relate 'flare-ups' to the same biomedical factors.

A number of participants told stories of structural and biomedical beliefs becoming deep-rooted and established when reinforced. For example, one participant recounted multiple encounters with healthcare practitioners that influenced and reinforced her structural belief.

"The work physio guy said to me that he thinks that my heels have maybe gone in which has then pulled my kneecap out of alignment. So instead of going smoothly over the joint where it's supposed to, that it's probably moving over the bone and that's the sharp pain that I'm feeling. Which did make sense because it, like I said, felt like I'd got a rock underneath my kneecap at some stage." [P9].

Some participants remembered biomechanical focused diagnoses they had been given by a healthcare practitioner they had seen many years in the past; highlighting the power and lasting influence healthcare practitioners have on their patients. For example one participant remembered the diagnosis she had received from a healthcare practitioner over 10 years ago:

"I had to go to the hospital once to have x-rays… I don't know if he [doctor] was trying to scare me into doing some exercise or something, but he basically said the only thing they could do is break both of my thighs and twist them a bit and then heal them back together. And it would take me years to get back to walking properly." [P4].

Joint noises are a common feature of normal joint movement,[22] however participants commonly reported distress and confusion at joint noises, often finding healthcare practitioners' explanations inadequate.

"It was the noise that was concerning me more than the pain. I'm used to hurting. I'm too small to play rugby for a start, and I'd been fighting for 20 years, so, erm, it's one of those, you get used to the pain, but it's just the noise. When you start, you sort of [say] no, that's not right." [P3].

This was in agreement with previous research, which identified negative emotions and inaccurate etiological beliefs with joint noises in patients with PFP.[37]

For peer review only

Expressly linked to participants' confusion and need to find the cause of their pain was also a strong desire to pursue radiological imaging, and feelings of not being taken fully seriously by the healthcare profession when this was not forthcoming.

"I want to know exactly what the problem is. Obviously, the doctor said, previously going back, they said tendonitis, and now they're saying it's runner's knee or whatever. But you know, it's still like, is that 100%, are you sure that's what it is? Because I was going to ask the doctor to send me for a MRI..." [P8].

Previous research has linked poor outcomes with radiological imaging in populations with low back pain, suggesting an over use of imaging has a detrimental effect on outcomes.[38] There was one example of the resulting radiological findings compounding the confusion and distrust, for example Participant Six explained her feelings on a normal MRI finding as:

"I mean I was a bit concerned, because they didn't turn around and say, you have hurt it, but it's not major but this is what you've done, but they didn't actually, they said nothing's wrong, take the knee brace off, and carry on. [I was] almost deflated, because I was like wanting to know why it was hurting, but they weren't explaining any of that to me. So it's a bit like, difficult." [P6].

Another participant's story demonstrates the negative impact of discordance between healthcare practitioners' diagnosis and advice, further compounding confusion and mistrust:

"Well, it makes you wonder then which one to believe, because I'm like, 'Well okay, he's told me not to do anything until I'm pain-free, because he doesn't want me to aggravate it,' but when, when I came here, and obviously they said that it would probably be best to start putting an impact on it again … " [P9].

The sense-making processes that participants described were established from past experience of healthcare treatment, past experience of pain and cultural beliefs around structure and pain.

Theme 3: exercise and activity beliefs

All participants identified specific beliefs regarding barriers to exercise and activity. These were informed by factors relating to: diagnosis uncertainty; cultural beliefs around pain; fear-avoidant behaviours and the iatrogenic effect of healthcare.

Diagnosis uncertainty, contributed to participants' beliefs regarding exercise and activity. In particular, it underpinned a dilemma regarding the relationship between activity and potential harm:

"It's 'are you making it worse?' And that's the crux of it really. As I'm doing it and thinking, 'if this is hurting, should I really be doing this, or shall I pack this in and do something else?' But it's the not knowing …" [P5].

Cultural beliefs around pain being a direct sign of tissue damage was evident in a large proportion of the participants' narratives, resulting in negative behaviour towards exercise and activity.

"…with me it's always been, if something hurt it because your body's telling you if you do that you're going to cause more injury. You'll make things worse." [P6].

For peer review only

Associated with the cultural beliefs on pain and damage was the resultant fear-avoidant behaviour. Participants, frequently contradicted themselves however; many participants would express the sentiment that they would not let the pain stop them from doing what they wanted to do, yet demonstrated clear activity withdrawal.

> *"So for example, we went to [holiday resort] last year; on your feet all day, walking miles and miles, I would be, like, in tears by the end of the day. I wouldn't let it stop me the next day because I would be, like, I'm doing this" [P4].*
>
> *"When I was in [holiday resort]; a couple of days I didn't go out and I stayed back at the hotel. Because I couldn't do it, I needed to rest." [P4].*

A predominant sub-theme was the association of sport and exercise, even in the absence of pain, as a potential precursor to future joint pain and 'damage'. Some participants attributed their current PFP to past sporting activities, despite no obvious mechanisms of injury.

> *"Yeah. Obviously it stems from doing long distance running." [P7].*

A number of participants discussed the direct impact of healthcare practitioner's advice and diagnosis labelling on their exercise and activity levels, suggesting an iatrogenic effect of healthcare for PFP patients.

> *"I have been told by doctors before I shouldn't run because it would jar my knee and shouldn't run or walk on an uneven surface because it will wonk my knee from side to side." [P4].*

> *"But then when I started the physio at work and he told me that I shouldn't walk or that I shouldn't swim because he just wanted to obviously manipulate it and get me pain-free before I did anything that could possibly aggravate it. So I stopped." [P9].*

This theme identified a number of beliefs associated as a barrier to activity and exercise engagement. These included diagnosis uncertainty; cultural beliefs around pain; fear-avoidant behaviours and the iatrogenic effect of healthcare.

Theme 4: behavioural coping strategies

A central coping strategy for participants of this study was the concept of rest. Many of them associated rest, and avoidance of activity, with the idea that time was necessary for the healing process, and that aggravating activities should be avoided.

> *"I try, obviously, sit down as much as I can." [P4].*

One participant expressed an expectation that healthcare professionals would advise him not to continue with activity and exercise:

> ***R:** So you think physios would say no [to keep physically active]?*

For peer review only - http://bmjopen.bmj.com/site/about/guidelines.xhtml

***P8:** Physios would probably say no. Yeah, you shouldn't do it.*

Another common coping strategy was postural adjustments; participants often talked of preferred sitting positions in relation to knee flexion.

In keeping with previous research on the high levels of analgesic use in patients with PFP,[7] a common narrative shared with participants was the use of analgesics, with some acknowledging they were not effective.

"I have had some strong painkillers from the doctors. They gave me some naproxen and some codeine to manage it when it was at its worst but I try not to take them." [P9].

The use of knee supports was also common in the self-management strategies employed by the participants.

"If it hurts, it hurts. I'll try and strap my knee up. Because if I know I'm going harder in like gym classes, I'll strap my knees up before I go. And then when I get too much pain, I'll stop the exercise." [P10].

Theme 5: expectations of the future

A number of participants expressed views, which could be contextualised as an external locus of control, with expectations of passive physiotherapeutic treatment options.

"I would presume manipulation of muscles groups, joints and tendons." [P3].

Even though the majority of participants expressed negative views about the future, they all expressed a desire to be pain free, over and above any functional improvements.

***R:** With the physio, what would you class as a success?*

***P8:** Getting rid of the pain.*

Nine of the ten participants held negative beliefs about the future; particularly in relation to prognostic prediction following their referral to physiotherapy.

"But then when I'm going up the stairs and it hurts it does concern me that it's going to be every day for the rest of my life I'm going to be struggling to walk upstairs. And then I think about getting old, and I think I'm going to end up with a stair lift and living downstairs and that sort of thing." [P1].

" [the pain is] definitely preying on my mind. Is it gonna stop me from going into the police, is that gonna stop me doing the things I want to do later on in life? So yeah, it does prey on my mind a little bit." [P6].

Central to their negative beliefs about the future and their prognosis was low self-efficacy. Participants felt they had very little control over their symptoms.

For peer review only

"[In] my head, my thought process is I just hate it. Do an operation. Get rid of it. In my head, and obviously not being from the medical profession, but I'm just like, "Just get rid of the pain however it can be done." [P8].

"Yes, I'm 37 now and they feel older than that. You just get that feeling, don't you, I've bounced back from lots of injuries before but this is the one that is making me think. You know, when this gets cold I can feel it, and thinking there's already arthritis there, I'm in trouble, it sets the brain going." [P3].

Low expectation of physiotherapy, and past physiotherapy failed treatments were also a core theme within future expectations.

R: *Have you got any expectations of what might happen when you walk in to see the physio?*

P10: *I expect them to turn around and say physio can't help.*

"When I did get the physiotherapy it kinda didn't really do anything anyway. So it just made me think, it's pointless, 'cause they was trying to remove the fluid from out my knee, that like I say, made it worse to begin with. She did say your knees will feel sore, but it went back to how it was anyway, so, it just seemed like a pointless process." [P7].

There was one exception, with one participant having positive outlook to the future and their physiotherapy referral.

"Oh yeah, I think it will get better. Yeah, I'd go for the better option." [P9].

The main sub-themes that emerged under the future were: beliefs that their pain will get worse; external locus of control with regards to treatment; low self-efficacy; poor opinion of physiotherapy and previous failed physiotherapy treatments and an overwhelming desire to be pain free, over and above any practical goals for rehabilitation.

Discussion

Main Findings

Quantitative research methodologies dominate the literature for PFP. This is the first study to use a qualitative method of inquiry to gain data on the experiences of people living with PFP. The five major themes that emerged from the data were: (1) impact on self; (2) uncertainty, confusion and sense making; (3) exercise and activity beliefs; (4) behavioural coping strategies and (5) expectations of the future.

A key finding of this study is that loss of physical ability is profound and considerable, and plays a significant role in participants' lives; despite previous research suggesting that PFP is a benign and

For peer review only

self-limiting condition.[5] An inability to continue with significant and meaningful activities has been identified as a cause of anxiety in people with persistent pain.[39] Persistent pain interrupts behaviour and a person's self-identity by affecting a sense of who they are, and what they might become.[40] As a result, lives are socially and environmentally restricted by persistent interruptions, or an inability to complete, or even attempt important tasks and activities.[40] With changes and loss of participants' position and role, for example with employment or family duties, the internalised meanings and expectations associated with one's self-identity is further threatened.[32]

Participants expressed intense confusion around their pain and symptoms. For instance, the causative reasons were elusive and troubling, as too was the ability to predict and control the pain intensity; and any attempts that participants made at understanding were firmly within the biomechanical sphere of reasoning. An inability to make sense of pain, and the process associated with sense-making and pain-related fear has been proposed in low back pain populations.[41] Previous research has identified that an inability to make sense of pain places 'lives on hold',[42] and may lead to more 'catastrophising'.[43]

There remains scientific debate and uncertainty around the underlying aetiology of PFP,[44] and there is a large variation in the way PFP is managed by physiotherapists in the UK.[45] The majority of participants in this study had previous experience of healthcare management for PFP suggesting that variation in healthcare treatment may have a negative impact on the patients' lived experience. Historically the biomedical model of pain establishes a direct relationship between tissue structure and pain,[46] and participants characteristically attributed their pain to structure and/or anatomical problems. However several studies have recently demonstrated that structural abnormalities of the patellofemoral joint on Magnetic Resonance Imaging (MRI) are not associated with PFP.[47,48] Three participants had no previous healthcare management for PFP, but nevertheless gave a biomechanical/structural cause for their pain; all three had previous physiotherapy for other pain conditions, including back, hips and ankles. This may suggest that exposure to biomechanical approaches to the management of musculoskeletal pain in general could, potentially, have a carryover to other locations of pain, with a negative effect.

The iatrogenic effect of healthcare is an emerging field of research in the low back pain population [38,49]. This study is the first to find such a theme in patients with PFP. These findings are consistent with recent research that showed that the majority of UK physiotherapists would advise their patients not to continue with exercises if they experienced any pain.[45] The fear-avoidance model of pain is a well-established with patients with persistent pain, particularly persistent low back pain;[17] additionally research has shown that fear-avoidance behaviour may also exist with clinicians.[25,45,50] The central concept of the model is cognitions and emotions that underpin fear of the pain; fears about potential physical activities exacerbating the pain and further 'damaging' bodies. The fear leads to safety seeking behaviours and hypervigilance that paradoxically maintains or exacerbates the pain and disability.[22] In contrast, if pain is perceived in a non-threatening way patients are likely to maintain physical activity levels, through which recovery can be achieved.[51,52] All of the ten participants in this study described fear-avoidant behaviour at some stage of their interview. This is the first study, which we know of, that identifies this behaviour in patients with a diagnosis of PFP.

Patellofemoral pain is often described as an 'overuse' injury,[53] and these data seem to be consistent with the patients' belief and behaviour with a definition more aligned with the English language meaning of 'overuse'. Contemporary thinking in relation to injury risk challenges the idea that PFP is

simply an 'overuse' injury, with evidence suggesting that persistent and long-term under-use may be a risk factor, with consistent exposure to tissue load being considered one method of management.[54] The fear-avoidant behaviours revealed within this study would therefore be seen as negative pain behaviour, with long-term detrimental consequences.

A key finding of this research is the low expectation for the future and low self-efficacy demonstrated by the majority of the participants that could be conceptualised as 'catastrophising'. Catastrophising is conceptually within the same model of pain behaviour as fear-avoidance, with largescale overlap.[19] Low self-efficacy, fear of the future and catastrophising is a common finding in patients with persistent pain.[24,55] The National Institute of Health and Care Excellence describes pain as a complex biopsychosocial issue, associated with expectations, self-efficacy, mood and coping abilities.[56] In addition, it has been shown that self-efficacy is a strong predictor of successful outcome, irrespective of the intervention delivered, for patients with persistent pain; suggesting that rehabilitation programmes for persistent musculoskeletal pain should be designed with the aim of improving self-efficacy.[57]

Clinical and research implications

This study established that a sample of patients with PFP demonstrated: pain-related fear, such as fear-avoidance; damage beliefs; difficulty with making sense of their pain; low self-efficacy and fear of the future.

The current consensus that best evidence treatments consisting of hip and knee strengthening may not be adequate to address the fears and beliefs identified in the current study. Future studies are needed to explore biopsychosocial targeted interventions for this population, particularly in relation to pain experienced by patients during exercise, followed by efficacy and effectiveness trials. Interventions may be patient education packages and self-management strategies targeting self-efficacy and physical activity. Furthermore, future qualitative work will be beneficial to understand the role of medical terminology commonly used with this patient group, for example, 'weakness' and 'patellar mal-tracking',[45] and its impact and interpretation by patients.

Study limitations and strengths

Two authors independently coded all transcripts, and this study employed a clear, transparent and reproducible methodological approach to data analysis. The authors make it clear that their clinical and research experience lie within the biopsychosocial framework of musculoskeletal pain and this study forms part of a larger body of research looking at pain education, self-management strategies and exercise interventions for individuals with PFP.[58] It is worth noting that the interviewer made it explicit to the participants that he was a physiotherapist working in the department conducting the research; indeed a number of them did proceed to ask clinical questions about their condition, highlighting a power dynamic between the interviewer and participant. Furthermore, it is important to note that recruitment took place in the same department that the researcher was working as a physiotherapist. This may, is part, have influenced participants' inclination to take part, and also their responses.

The main limitation of this study is that for pragmatic reasons a convenience sampling technique was used. It is possible that this sample may differ from other samples within the UK, and how representative these findings are to the greater population of individuals with PFP is unknown. A

For peer review only - http://bmjopen.bmj.com/site/about/guidelines.xhtml

purposive sampling technique may have better represented sociodemographic groups, or targeted identifiable subgroups.

Conclusion

These findings offer an insight into the experience of individuals living with PFP. Previous literature have focused on pain and biomechanics, rather than the individual experience, attached meanings and any wider context within a sociocultural perspective. The participants provided rich and detailed narratives of loss of physical and functional ability; loss of self-identity; pain related confusion and difficulty making sense of their pain; pain-related fear, including fear-avoidance and 'damage' beliefs; inappropriate coping strategies and fear of the future. Our findings suggest future research is warranted into biopsychosocial targeted interventions and the impact and interpretation of medical terminology.

Authors' contributions

BES was responsible for conception and design, compiling the interview schedule, interviewing, transcribing, coding, analysis and interpretation, drafting and revising the manuscript. FM was responsible for conception and design, compiling the interview schedule, coding, analysis and interpretation, drafting and revising the manuscript. PH, MB, JS, MR, TS and PL were involved in conception and design, interpretation and reviewing revisions to the manuscript. All authors have read and approved of the final manuscript.

Funding

This report is independent research arising from a Clinical Doctoral Research Fellowship, Benjamin Smith, ICA-CDRF-2015-01-002 supported by the National Institute for Health Research (NIHR) and Health Education England (HEE). The views expressed in this publication are those of the author(s) and not necessarily those of the NHS, the NIHR, HEE or the Department of Health.

Competing interests

The authors declare that they have no competing interests.

Ethics approval

This study was approved by the West Midlands - Black Country Research Ethics Committee (16/WM/0414).

Availability of data

Quotations and further details are available from Benjamin Smith at benjamin.smith3@nhs.net

Supplementary Data

Data supplement 1 – Code Book

For peer review only

References

1 Dey P, Callaghan M, Cook N, *et al.* A questionnaire to identify patellofemoral pain in the community: an exploration of measurement properties. *BMC Musculoskelet Disord* 2016;17:237. doi:10.1186/s12891-016-1097-5

2 Urwin M, Symmons D, Allison T, *et al.* Estimating the burden of musculoskeletal disorders in the community: the comparative prevalence of symptoms at diVerent anatomical sites, and the relation to social deprivation. *Ann Rheum Dis* 1998;57:649–55.

3 Jordan KP, Kadam UT, Hayward R, *et al.* Annual consultation prevalence of regional musculoskeletal problems in primary care: an observational study. *BMC Musculoskelet Disord* 2010;11:144. doi:10.1186/1471-2474-11-144

4 Crossley KM, Stefanik JJ, Selfe J, *et al.* 2016 Patellofemoral pain consensus statement from the 4th International Patellofemoral Pain Research Retreat, Manchester. Part 1: Terminology, definitions, clinical examination, natural history, patellofemoral osteoarthritis and patient-reported outcome m. *Br J Sports Med* 2016;50:839–43.

5 Sandow MJ, Goodfellow JW. The natural history of anterior knee pain in adolescents. *J Bone Joint Surg Br* 1985;67:36–8.

6 Collins NJ, Bierma-Zeinstra SMA, Crossley KM, *et al.* Prognostic factors for patellofemoral pain: a multicentre observational analysis. *Br J Sports Med* 2013;47:227–33. doi:10.1136/bjsports-2012-091696

7 Stathopulu E, Baildam E. Anterior knee pain: a long-term follow-up. *Rheumatology (Oxford)* 2003;42:380–2.

8 Rathleff MS, Rasmussen S, Olesen JL. Unsatisfactory long-term prognosis of conservative treatment of patellofemoral pain syndrome. *Ugeskr Laeger* 2012;174:1008–13.

9 Smith BE, Hendrick P, Logan P. Patellofemoral pain: Challenging current practice - A case report. *Man Ther* 2016;22:216–9. doi:10.1016/j.math.2015.09.002

10 Doménech J, Sanchis-Alfonso V, López L, *et al.* Influence of kinesiophobia and catastrophizing on pain and disability in anterior knee pain patients. *Knee Surgery, Sport Traumatol Arthrosc* 2013;21:1562–8. doi:10.1007/s00167-012-2238-5

11 Piva SR, Fitzgerald GK, Wisniewski S, *et al.* Predictors of pain and function outcome after rehabilitation in patients with patellofemoral pain syndrome. *J Rehabil Med* 2009;41:604–12. doi:10.2340/16501977-0372

For peer review only

12 Piva SR, Fitzgerald GK, Irrgang JJ, *et al.* Associates of Physical Function and Pain in Patients with Patellofemoral Pain Syndrome. *Arch Phys Med Rehabil* 2009;90:285–95. doi:10.1016/j.apmr.2008.08.214

13 Quartana PJ, Campbell CM, Edwards RR. Pain catastrophizing: a critical review. *Expert Rev Neurother* 2009;9:745–58. doi:10.1586/ern.09.34

14 Moseley GL, Arntz A. The context of a noxious stimulus affects the pain it evokes. *PAIN®* 2007;133:64–71.

15 Harvie DS, Broecker M, Smith RT, *et al.* Bogus visual feedback alters onset of movement-evoked pain in people with neck pain. *Psychol Sci* 2015;:0956797614563339.

16 Lobanov O V, Zeidan F, McHaffie JG, *et al.* From cue to meaning: Brain mechanisms supporting the construction of expectations of pain. *PAIN®* 2014;155:129–36.

17 Chou R, Shekelle P. Will this patient develop persistent disabling low back pain? *JAMA* 2010;303:1295–302. doi:10.1001/jama.2010.344

18 Mallows A, Debenham J, Walker T, *et al.* Association of psychological variables and outcome in tendinopathy: a systematic review. *Br J Sports Med* 2016;:bjsports – 2016.

19 Wertli MM, Rasmussen-Barr E, Weiser S, *et al.* The role of fear avoidance beliefs as a prognostic factor for outcome in patients with nonspecific low back pain: a systematic review. *spine J* 2014;14:816–36.

20 Osborn M, Smith JA. The personal experience of chronic benign lower back pain: An interpretative phenomenological analysis. *Br J Pain* 2015;9:65–83.

21 Smith JA, Osborn M. Pain as an assault on the self: An interpretative phenomenological analysis of the psychological impact of chronic benign low back pain. *Psychol Heal* 2007;22:517–34.

22 Jones S, Hanchard N, Hamilton S, *et al.* A qualitative study of patients' perceptions and priorities when living with primary frozen shoulder. *BMJ Open* 2013;3:e003452.

23 Walker J, Holloway I, Sofaer B. In the system: the lived experience of chronic back pain from the perspectives of those seeking help from pain clinics. *Pain* 1999;80:621–8.

24 Walker J, Sofaer B, Holloway I. The experience of chronic back pain: accounts of loss in those seeking help from pain clinics. *Eur J Pain* 2006;10:199.

For peer review only

25 Nijs J, Roussel N, Paul van Wilgen C, *et al.* Thinking beyond muscles and joints: Therapists' and patients' attitudes and beliefs regarding chronic musculoskeletal pain are key to applying effective treatment. *Man Ther* 2013;18:96–102.

26 Thorne S, Kirkham SR, MacDonald-Emes J. Focus on qualitative methods. Interpretive description: a noncategorical qualitative alternative for developing nursing knowledge. *Res Nurs Health* 1997;20:169–77.

27 Braun V, Clarke V. Using thematic analysis in psychology. *Qual Res Psychol* 2006;3:77–101.

28 Tong A, Sainsbury P, Craig J. Consolidated criteria for reporting qualitative research (COREQ): a 32-item checklist for interviews and focus groups. *Int J Qual Heal care* 2007;19:349–57.

29 Mc Auliffe S, Synott A, Casey H, *et al.* Beyond the tendon: Experiences and perceptions of people with persistent Achilles tendinopathy. *Musculoskelet Sci Pract* 2017;29:108–14.

30 Leary MR, Tangney JP. *Handbook of self and identity*. Guilford Press 2011.

31 Baumeister RF. *The self in social psychology*. 1999.

32 Stryker S, Burke PJ. The past, present, and future of an identity theory. *Soc Psychol Q* 2000;:284–97.

33 Lamé IE, Peters ML, Vlaeyen JWS, *et al.* Quality of life in chronic pain is more associated with beliefs about pain, than with pain intensity. *Eur J Pain* 2005;9:15–24.

34 Aldrich S, Eccleston C. Making sense of everyday pain. *Soc Sci Med* 2000;50:1631–41.

35 Charmaz K. From the' sick role' to stories of self: Understanding the self in illness. *Rutgers Ser Self Soc Identity* 1999;2:209–39.

36 Johansson EE, Hamberg K, Westman G, *et al.* The meanings of pain: an exploration of women's descriptions of symptoms. *Soc Sci Med* 1999;48:1791–802.

37 Robertson CJ, Hurley M, Jones F. People's beliefs about the meaning of crepitus in patellofemoral pain and the impact of these beliefs on their behaviour: A qualitative study. *Musculoskelet Sci Pract* 2017;28:59–64. doi:10.1016/j.msksp.2017.01.012

38 Webster BS, Bauer AZ, Choi Y, *et al.* Iatrogenic consequences of early magnetic resonance imaging in acute, work-related, disabling low back pain. *Spine (Phila Pa 1976)* 2013;38:1939–46.

For peer review only

39 Roy R. *Chronic pain, loss and suffering*. University of Toronto Press 2004.

40 Harris S, Morley S, Barton SB. Role loss and emotional adjustment in chronic pain. *Pain* 2003;105:363–70.

41 Bunzli S, Smith A, Schütze R, *et al.* Beliefs underlying pain-related fear and how they evolve: a qualitative investigation in people with chronic back pain and high pain-related fear. *BMJ Open* 2015;5.

42 Bunzli S, Watkins R, Smith A, *et al.* Lives on hold: a qualitative synthesis exploring the experience of chronic low-back pain. *Clin J Pain* 2013;29:907–16.

43 van Wilgen CP, van Ittersum MW, Kaptein AA, *et al.* Illness perceptions in patients with fibromyalgia and their relationship to quality of life and catastrophizing. *Arthritis Rheum* 2008;58:3618–26.

44 Doménech J, Sanchis-Alfonso V, Espejo BBB, *et al.* Changes in catastrophizing and kinesiophobia are predictive of changes in disability and pain after treatment in patients with anterior knee pain. *Knee Surg Sports Traumatol Arthrosc* 2014;22:2295–300. doi:10.1007/s00167-014-2968-7

45 Smith BE, Hendrick P, Bateman M, *et al.* Current Management Strategies for Patellofemoral Pain: An online survey of 99 practising UK physiotherapists. *BioMed Cent Musculoskelet Disord* 2017;18. doi:10.1186/s12891-017-1539-8

46 Doménech J, Sanchis-Alfonso V, Espejo B. Influence of Psychological Factors on Pain and Disability in Anterior Knee Pain Patients. In: *Anterior Knee Pain and Patellar Instability*. London: : Springer London 2011. 123–35. doi:10.1007/978-0-85729-507-1_9

47 van der Heijden RA, Oei EHG, Bron EE, *et al.* No Difference on Quantitative Magnetic Resonance Imaging in Patellofemoral Cartilage Composition Between Patients With Patellofemoral Pain and Healthy Controls. *Am J Sports Med* 2016;44:1172–8. doi:10.1177/0363546516632507

48 van der Heijden RA, de Kanter JLM, Bierma-Zeinstra SMA, *et al.* Structural Abnormalities on Magnetic Resonance Imaging in Patients With Patellofemoral Pain: A Cross-sectional Case-Control Study. *Am J Sports Med* 2016;44:2339–46. doi:10.1177/0363546516646107

49 Lin IB, O'Sullivan PB, Coffin JA, *et al.* Disabling chronic low back pain as an iatrogenic disorder: a qualitative study in Aboriginal Australians. *BMJ Open* 2013;3:1–8.

50 Littlewood C, Lowe A, Moore J. Rotator cuff disorders: a survey of current UK physiotherapy practice. *Shoulder Elb* 2012;4:64–71. doi:10.1111/j.1758-5740.2011.00164.x

For peer review only

51 Leeuw M, Goossens MEJB, Linton SJ, *et al.* The fear-avoidance model of musculoskeletal pain: current state of scientific evidence. *J Behav Med* 2007;30:77–94. doi:10.1007/s10865-006-9085-0

52 Vlaeyen JW, Linton SJ. Fear-avoidance and its consequences in chronic musculoskeletal pain: a state of the art. *Pain* 2000;85:317–32.

53 Herrington L, Nester C. Q-angle undervalued? The relationship between Q-angle and medio-lateral position of the patella. *Clin Biomech (Bristol, Avon)* 2004;19:1070–3. doi:10.1016/j.clinbiomech.2004.07.010

54 Gabbett TJ. The training-injury prevention paradox: should athletes be training smarter and harder? *Br J Sports Med* 2016;50:273–80. doi:10.1136/bjsports-2015-095788

55 Arnstein P, Caudill M, Mandle CL, *et al.* Self efficacy as a mediator of the relationship between pain intensity, disability and depression in chronic pain patients. *Pain* 1999;80:483–91.

56 National Institute for Clinical Excellence. Osteoarthritis: care and management. 2014.

57 Miles CL, Pincus T, Carnes D, *et al.* Can we identify how programmes aimed at promoting self-management in musculoskeletal pain work and who benefits? A systematic review of sub-group analysis within RCTs. *Eur J Pain* 2011;15. doi:10.1016/j.ejpain.2011.01.016

58 Smith BE, Hendrick P, Bateman M, *et al.* Study protocol: a mixed methods feasibility study for a loaded self-managed exercise programme for patellofemoral pain. *Pilot Feasibility Stud* 2017;4. doi:10.1186/s40814-017-0167-2

For peer review only

Data supplement 1 – Code Book

Name

Impact on self, sense of loss

Name
Pain experiences, disruption to life, distressing
Loss of identity, self
Relationships loss (time with family, friends, children, friends making fun, judgement)
Knee noises experiences, distressing

Not understanding, sense making, confusion

Name
Trying to find cause
Anatomy and imaging central to causation
Historical diagnosis – i.e historical comments still central to beliefs
Distrust of healthcare – wanting scans
Not being taken seriously
Disagreement with healthcare increases uncertainty and distress

Exercise and Activity Barriers

Name
Diagnosis uncertainty
Cultural beliefs around pain
Fear avoidance
Iatrogenic effect of healthcare
Sport = future 'damage'
Physio = exercise
contradiction

Coping Strategies

Name
Central to coping is activity avoidance – despite saying they don't let it stop them
Postural adjustments
Rest
Analgesics – whilst acknowledging they don't work
Reliance on knee support – not knowing how they work

The Future

Name
Belief will get worse
Effect of healthcare on that belief (including external locus of control)
Low self-efficacy
Low opinion of physio, past physio failures
Desire to be pain free
high expectation of prognosis

For peer review only

Consolidated criteria for reporting qualitative studies (COREQ): 32-item checklist

Developed from:
Tong A, Sainsbury P, Craig J. Consolidated criteria for reporting qualitative research (COREQ): a 32-item checklist for interviews and focus groups. *International Journal for Quality in Health Care*. 2007. Volume 19, Number 6: pp. 349 – 357

YOU MUST PROVIDE A RESPONSE FOR ALL ITEMS. ENTER N/A IF NOT APPLICABLE

No. Item	Guide questions/description	Reported on Page #
**Domain 1: Research team and reflexivity**		
Personal Characteristics		
1. Inter viewer/facilitator	Which author/s conducted the inter view or focus group?	Page 5
2. Credentials	What were the researcher's credentials? E.g. PhD, MD	Page 5
3. Occupation	What was their occupation at the time of the study?	Page 5
4. Gender	Was the researcher male or female?	Page 5
5. Experience and training	What experience or training did the researcher have?	Page 5
Relationship with participants		
6. Relationship established	Was a relationship established prior to study commencement?	Page 5
7. Participant knowledge of the interviewer	What did the participants know about the researcher? e.g. personal goals, reasons for doing the research	Page 5
8. Interviewer characteristics	What characteristics were reported about the inter viewer/facilitator? e.g. Bias, assumptions, reasons and interests in the research topic	Page 5 & 15
**Domain 2: study design**		
Theoretical framework		
9. Methodological orientation and Theory	What methodological orientation was stated to underpin the study? e.g. grounded theory, discourse analysis, ethnography, phenomenology, content analysis	Page 5
Participant selection		
10. Sampling	How were participants selected? e.g. purposive, convenience, consecutive, snowball	Page 5
11. Method of approach	How were participants approached? e.g. face-to-face, telephone, mail, email	Page 5
12. Sample size	How many participants were in the study?	Page 5

For peer review only

13. Non-participation	How many people refused to participate or dropped out? Reasons?	Page 5
Setting		
14. Setting of data collection	Where was the data collected? e.g. home, clinic, workplace	Page 5
15. Presence of non-participants	Was anyone else present besides the participants and researchers?	Page 5
16. Description of sample	What are the important characteristics of the sample? e.g. demographic data, date	Page 7
Data collection		
17. Interview guide	Were questions, prompts, guides provided by the authors? Was it pilot tested?	Page 5
18. Repeat interviews	Were repeat inter views carried out? If yes, how many?	N/A
19. Audio/visual recording	Did the research use audio or visual recording to collect the data?	Page 5
20. Field notes	Were field notes made during and/or after the inter view or focus group?	Page 5 & 6
21. Duration	What was the duration of the inter views or focus group?	Page 7
22. Data saturation	Was data saturation discussed?	Page 6
23. Transcripts returned	Were transcripts returned to participants for comment and/or correction?	N/A
**Domain 3: analysis and findings**		
Data analysis		
24. Number of data coders	How many data coders coded the data?	Page 6
25. Description of the coding tree	Did authors provide a description of the coding tree?	N/A
26. Derivation of themes	Were themes identified in advance or derived from the data?	Page 6
27. Software	What software, if applicable, was used to manage the data?	NVivo
28. Participant checking	Did participants provide feedback on the findings?	NO
Reporting		
29. Quotations presented	Were participant quotations presented to illustrate the themes/findings? Was each quotation identified? e.g. participant number	Results
30. Data and findings consistent	Was there consistency between the data presented and the findings?	Page 13
31. Clarity of major themes	Were major themes clearly presented in the findings?	RESULTS
32. Clarity of minor themes	Is there a description of diverse cases or discussion of minor themes?	Discussion

Once you have completed this checklist, please save a copy and upload it as part of your submission. When requested to do so as part of the upload process, please select the file type: *Checklist*. You will NOT be able to proceed with

For peer review only

submission unless the checklist has been uploaded. Please DO NOT include this checklist as part of the main manuscript document. It must be uploaded as a separate file.

For peer review only
